# *Histoplasma capsulatum* Glycans From Distinct Genotypes Share Structural and Serological Similarities to *Cryptococcus neoformans* Glucuronoxylomannan

**DOI:** 10.3389/fcimb.2020.565571

**Published:** 2021-01-08

**Authors:** Diego de Souza Gonçalves, Claudia Rodriguez de La Noval, Marina da Silva Ferreira, Leandro Honorato, Glauber Ribeiro de Sousa Araújo, Susana Frases, Claudia Vera Pizzini, Joshua D. Nosanchuk, Radames J. B. Cordero, Marcio L. Rodrigues, José Mauro Peralta, Leonardo Nimrichter, Allan J. Guimarães

**Affiliations:** ^1^ Laboratório de Bioquímica e Imunologia das Micoses, Departamento de Microbiologia e Parasitologia, Instituto Biomédico, Universidade Federal Fluminense, Niterói, Brazil; ^2^ Pós-Graduação em Doenças Infecciosas e Parasitárias, Faculdade de Medicina, Universidade Federal do Rio de Janeiro, Rio de Janeiro, Brazil; ^3^ Departamento de Microbiologia Geral, Instituto de Microbiologia Paulo de Góes, Universidade Federal do Rio de Janeiro, Rio de Janeiro, Brazil; ^4^ Departamento de Imunologia, Instituto de Microbiologia Paulo de Góes, Universidade Federal do Rio de Janeiro, Rio de Janeiro, Brazil; ^5^ Laboratório de Biofísica de Fungos, Instituto de Biofísica Carlos Chagas Filho, Universidade Federal do Rio de Janeiro, Rio de Janeiro, Brazil; ^6^ Laboratório de Micologia, Instituto Nacional de Infectologia Evandro Chagas, Fundação Oswaldo Cruz, Rio de Janeiro, Brazil; ^7^ Department of Microbiology and Immunology and Division of infectious Diseases, Albert Einstein College of Medicine of Yeshiva University, Bronx, NY, United States; ^8^ Department of Molecular Microbiology and Immunology, Johns Hopkins Bloomberg School of Public Health, Baltimore, MD, United States; ^9^ Instituto Carlos Chagas, Fundação Oswaldo Cruz (Fiocruz), Curitiba, Brazil

**Keywords:** cellular-attached glycans, extracellular glycans, GXM-like, *Histoplasma capsulatum*, *Cryptococcus neoformans*, pathogenesis

## Abstract

The cell wall is a ubiquitous structure in the fungal kingdom, with some features varying depending on the species. Additional external structures can be present, such as the capsule of *Cryptococcus neoformans* (*Cn*), its major virulence factor, mainly composed of glucuronoxylomannan (GXM), with anti-phagocytic and anti-inflammatory properties. The literature shows that other cryptococcal species and even more evolutionarily distant species, such as the *Trichosporon asahii, T. mucoides*, and *Paracoccidioides brasiliensis* can produce GXM-like polysaccharides displaying serological reactivity to GXM-specific monoclonal antibodies (mAbs), and these complex polysaccharides have similar composition and anti-phagocytic properties to cryptococcal GXM. Previously, we demonstrated that the fungus *Histoplasma capsulatum (Hc)* incorporates, surface/secreted GXM of *Cn* and the surface accumulation of the polysaccharide enhances *Hc* virulence *in vitro* and *in vivo*. In this work, we characterized the ability of *Hc* to produce cellular-attached (C-gly-*Hc*) and secreted (E-gly) glycans with reactivity to GXM mAbs. These C-gly-*Hc* are readily incorporated on the surface of acapsular *Cn* cap59; however, in contrast to *Cn* GXM, C-gly-*Hc* had no xylose and glucuronic acid in its composition. Mapping of recognized *Cn* GXM synthesis/export proteins confirmed the presence of orthologs in the *Hc* database. Evaluation of C-gly and E-gly of *Hc* from strains of distinct monophyletic clades showed serological reactivity to GXM mAbs, despite slight differences in their molecular dimensions. These C-gly-*Hc* and E-gly-*Hc* also reacted with sera of cryptococcosis patients. In turn, sera from histoplasmosis patients recognized *Cn* glycans, suggesting immunogenicity and the presence of cross-reacting antibodies. Additionally, C-gly-*Hc* and E-gly-*Hc* coated *Cn* cap59 were more resistant to phagocytosis and macrophage killing. C-gly-*Hc* and E-gly-*Hc* coated *Cn* cap59 were also able to kill larvae of *Galleria mellonella*. These GXM-like *Hc* glycans, as well as those produced by other pathogenic fungi, may also be important during host-pathogen interactions, and factors associated with their regulation are potentially important targets for the management of histoplasmosis.

## Introduction

Regardless of the species, all fungi possess a surrounding polysaccharide enriched cell wall that varies in composition and structural organization (Erwig and Gow, 2016; Gow et al., 2017). The main structure shared by several human pathogenic species is composed of an inner layer of chitin, a water-insoluble polymer of N-acetyl-glucosamine units linked by β-1,4-glycosidic bonds, and, just external to it, a layer of branched β-1,3- or β-1,6-glucans (Gow et al., 2017).

These fungal surface polysaccharides are pathogen-associated molecular patterns (PAMPs) that are efficiently recognized by pattern recognition receptors (PRRs) on the surface of innate immunity cells for the initiation of the immune response (Romani, 2011). Fungal β-1,3-glucan, the main content of the fungal cell wall, is recognized by Dectin-1 on the surface of macrophages and dendritic cells (Guimaraes et al., 2011; Gow et al., 2017). In turn, chitin is recognized by Toll-like receptor (TLR)- 2 (Shen et al., 2016) or indirectly mediate fungal recognition through Dectin-1 (Mora-Montes et al., 2011).

Differences in immune recognition among species are also given by specific surface components attached to these core layers, such as the pigment melanin in *Fonsecaea pedrosoi*, rodlets and galactomannans in *Aspergillus fumigatus*, glucuronoxylomannan (GXM) capsule in *Cryptococcus* sp. (Gow et al., 2017) and α-1,3-glucans in thermally dimorphic fungi, among others (Guimaraes et al., 2011; Gow et al., 2017; Ray and Rappleye, 2019). Despite mechanical protection for the fungal cells, all of these components are involved in the fungal escape of the immune response, since they all can shield cells from immune recognition (Rappleye et al., 2007; Erwig and Gow, 2016; Gow et al., 2017).

Among these, the capsular structure of *Cryptococcus* sp. has been extensively characterized and is considered one of the main virulence factors of the fungus. It is predominantly composed of two polysaccharides; GXM, the most abundant and having a wide range of dimensions and molecular weights, and glucuronoxylomannogalactan (GXMGal) fibers. These complex polysaccharides are synthesized by the incorporation of individually activated monosaccharide-nucleotides in secretory organelles, by the catalysis of multiple glycosyltransferases in the Golgi complex (Janbon, 2004; Yoneda and Doering, 2006; Zaragoza et al., 2009). Classical secretory pathways are involved in the secretion of GXM to the extracellular milieu, where they can be incorporated into the inner interface or external edge of the existing capsule at the cell surface (Yoneda and Doering, 2006). GXM attachment and correct capsule assembly are dependent on the presence of α-1,3-glucan, as mutants lacking this synthesis pathway have entirely compromised cell wall structure and display an acapsular phenotype (Reese and Doering, 2003; Reese et al., 2007).

Previous reports have characterized the capacity of fungi other than *Cryptococcus* sp. to efficiently incorporate GXM to their cell surfaces (Reese and Doering, 2003; Cordero et al., 2016). We have demonstrated that *Histoplasma capsulatum* (*Hc*) yeast cells promptly attach cellular and extracellular glycans of *Cryptococcus neoformans* (*Cn*) onto its cell wall *in vitro* and *in vivo*, which enhanced *Hc* virulence, by transferring the antiphagocytic, immune inhibitory and biofilm inducing properties of these cryptococcal polysaccharides to the newly encapsulated fungus (Cordero et al., 2016). As surface GXM anchoring by *Hc* was also dependent on α-1,3-glucan (Reese and Doering, 2003), this also raised the hypothesis that *H. capsulatum* shared similar structures on the cell surface and mechanisms for attaching carbohydrate fibers. This observation might pose an important mechanistic observation on the transfer of virulence factors among fungi and explain why patients with concomitant infections due to *Cn* and *Hc* generally have severe illnesses [reviewed in (Cordero et al., 2016)].

Besides *Cryptococcus* spp (Araujo et al., 2017), another *Basidiomycota* are also able to produce GXM-like molecules. *Trichosporon asahii* produces a functional GXM-like molecule with similar glycosyl composition to cryptococcal GXM that also manifests antiphagocytic activities (Fonseca et al., 2009). Recently, Zimbres et al. also described the production of GXM-like polysaccharides by *T. mucoides* (Zimbres et al., 2018). In the phylum *Ascomycota*, GXM-like structures have been identified in the dimorphic fungus *Paracoccidioides brasiliensis* (Albuquerque et al., 2012). These glycans are mainly composed of mannose and galactose, and traces of glucose, xylose, and rhamnose, and display a lower effective diameter relative to *Cn* GXM. Overall, as observed for *Trychosporum* sp., *P. brasiliensis* glycans react with a panel of mAbs to *Cn* GXM and are also incorporated by the cap59 acapsular mutant of *Cn*, forming a capsular-like structure and sharing the GXM-like antiphagocytic properties.

As *Hc* is able to attach *Cn* GXM to the yeast cell surface, we cannot discard the possibility that this fungus is also able to produce capsular or shed components with direct implications to fungal virulence, similar to those of *Cn, Hc* capsular material may be immunomodulatory. Notably, no comparative evaluation has been performed regarding the similarity of glycans shed by these two fungi. Herein, we aimed to molecularly characterize the cell-associated and secreted extracellular glycans of the fungus *Hc* by 1) determining the serological reactivity to mAbs raised to cryptococcal GXM to *Hc* glycans, 2) assessing glycan reactivity to serum from cryptococcosis and histoplasmosis patients, 3) defining the macromolecular structure and glycosyl composition of the glycans, and 4) determining the role of surface glycans during fungus interactions with phagocytic cells and 5) the role in pathogenesis in *Galleria mellonella* model. We found that these surface and extracellular glycans of *Hc* have similar functions to *Cn* GXM; hence, as with *Cn* and other GXM-like displaying fungi, *Hc* surface glycans may also be important for the immunomodulatory functions during the pathogenesis of histoplasmosis. Furthermore, the metabolic pathways of GXM-like production in *Hc* may also be novel targets for the development of new antifungal drugs and therapeutic strategies for the management of histoplasmosis.

## Methods

### Fungal Strains and Growth Conditions

*C. neoformans* (*Cn*) var. *grubii* serotype A strain H99 (ATCC 208821) and the acapsular mutant *Cn* cap59 (ATCC 34873, derivative of Serotype D strain B3501) were kept on Sabouraud agar plates (Sigma-Aldrich, San Luis, MO, EUA). Colonies were inoculated in Sabouraud broth and kept at 37°C for 24 h under shaking at 150 rpm. For experimental procedures, subcultures were performed in minimal media (MM, 29.4 mM KH_2_PO_4_, 10 mM MgSO_4_, 13 mM glycine, 3 µM thiamine and 15 mM D-glucose, pH 5.5) at 37°C for 48 h (Cordero et al., 2016). *H. capsulatum* (*Hc)* var. *capsulatum* strains from distinct monophyletic clades as established previously (Teixeira Mde et al., 2016; Sepulveda et al., 2017), *Hc* G217B (ATCC 26032), *Hc* G184A (ATCC 26027) and *Hc* CIB1980 (a kind gift from Corporación para Investigaciones Biológicas, Colombia) strains were cultured in HAM’s F-12 (ThermoFisher Scientific) medium supplemented with glucose (18.2 g/L), glutamic acid (1 g/L), HEPES (6 g/L), and cysteine (8.4 mg/L) at 37°C for 48 h with 150 rpm shaking (Guimarães et al., 2009).

### Isolation of Cellular-Attached and Extracellular Fungal Glycans

After 48 h, 1 L cultures of *Hc* G217B, *Hc* G184A and *Hc* CIB1980 or *Cn* H99 yeast cells were separately centrifuged for 10 min at 1100 x *g*. Cell pellet and cell-free culture supernatants were collected for the extraction of cellular-attached glycans (C-gly) and isolation of shed extracellular glycans (E-gly) respectively as described (Frases et al., 2008; Cordero et al., 2016). C-gly extraction was carried out by DMSO extraction. Briefly, yeast pellets obtained upon centrifugation were washed with PBS (8.0 g/L NaCl, 0.2 g/L KCl, 0.2 g/L KH_2_PO_4_, 1.2 g/L Na_2_HPO_4_, pH 7.2) and incubated in DMSO for 1 h with 150 rpm shaking and centrifuged. E-glys were obtained by ultrafiltration of the cell-free culture supernatant using an Amicon cell coupled with a nitrocellulose membrane with a nominal molecular weight limit (NMWL) of 10 KDa (Millipore, MA, USA) and N_2_. Concentrated C-gly and E-gly were dialyzed against MilliQ water for 24 h, using a 3.5 KDa cut-off dialysis tube with at least 8 water exchanges performed. Glycans were lyophilized using standard protocols. Polysaccharide (PS) concentration of C-gly and E-gly were measured by phenol-sulfuric acid method as described (Masuko et al., 2005).

### Binding of GXM mAbs to Cellular-Attached and Extracellular Glycans

ELISA plates were coated with 50 µl/well of a 10 µg/ml *Cn* or *Hc* glycans solution in PBS for 1 h at 37°C, followed by an overnight step at 4°C. Plates were washed 3X with TBS-T (8.0 g/L NaCl, 1.21 g/L Tris base, 0,01% Tween 20, pH 7.2) and blocked with blocking solution (1% BSA in TBS-T) for 1 h at 37°C. After washes with TBS-T, mAbs to GXM (Mukherjee et al., 1993), IgG1 18B7 or IgMs 2D10, 13F1, and 12A1 or irrelevant antibody 5C11 as a control at 25 µg/ml were serially diluted (1:2) in blocking solution across the plates and incubated at 37°C for 1 h. Wells were washed (3X) and incubated with goat anti-mouse Ig (Southern Biotech) at 1 μg/ml for 1 h at 37°C. After washes, plates were incubated with 1mg/ml of p-nitrophenylphosphate (pNPP, Sigma-Aldrich). Absorbances were recorded at 405 nm. Experiments were performed in triplicates and results shown are the average of three independent experiments.

### Incorporation of *Hc* C-gly by an acapsular mutant of *Cn*

Approximately, 5x10^6^ *Cn* cap59 yeasts were suspended in 50 µg/ml of C-gly preparations from *Hc* G217B (C-gly-*Hc*) reference strain (10 µg/10^6^ cells) (Cordero et al., 2016). The cell suspension was incubated for 1 h at 37°C under agitation and extensively washed with PBS to remove unbound glycans. As controls, *cap59* cells were incubated with C-gly-*Cn* H99 (positive) or PBS alone (negative) were used. Yeasts (10^6^) of *Hc* G217B were also used in parallel. After three washes with PBS and centrifugations at 1100 x *g* for 10 min, yeasts were suspended in 100 µl of a solution containing 10 µg/ml mAb 18B7 (IgG1) in 1% BSA in PBS and incubated for 1 h at 37°C in an orbital shaker. Following incubation, cells were washed (3X) with PBS and collected by centrifugation. Cell pellets were suspended in 100 µl of a 5 µg/ml solution of a goat anti-mouse IgG Alexa 488-conjugated (Southern Biotech) in blocking solution and incubated for 1 h at 37°C. After three washes with PBS, yeasts were stained using 0.5 mg/ml of Uvitex 2B (stains chitin in cell wall). Cells were washed, suspended in mounting solution (Biomeda Corp, Foster City, CA), applied to a microscopy slide, and examined in a Zeiss Axiovert 200 fluorescence inverted phase and contrast microscope using a 100X/1.30 Oil Plan Neofluar objective (Carl Zeiss MicroImaging, Inc.).

### Glycosyl Composition Analysis

C-gly preparations from *Hc* G217B and *Cn* H99 were lyophilized and analyzed at the Complex Carbohydrate Research Center (CCRC, Athens, GA, USA) as described (Guimaraes et al., 2010). Samples were dissolved in methanol/1M HCl, followed by incubation for 18 h at 80°C for methanolysis. Then, samples were per-O-trimethylsilylated with Tri-Sil (Pierce) for 30 min at 80°C. Derivatives were separated on a HP5890 gas-chromatography coupled with a Supelco DB-1 silica capillary column (30 m × 0.25 mm ID). Detected peaks were fragmented and detected in tandem with a 5,970 MSD mass spectrometer. Monosaccharide standards consisted of arabinose, dulcitol, fucose, galactose, galacturonic acid, glucose, glucuronic acid, mannitol, mannose, N-acetyl gucosamine, rhamnose, sorbitol and xylose. Molecular ratios were calculated by dividing the percentage of each carbohydrate measured in the sample by its respective molecular weight. The values reported are representative of two independent analysis using different sample batches, with similar results.

### Zeta Potential Analysis

Zeta potential (Z) of *Cn* H99 and acapsular mutants *Cn* cap 59 incubated with either PBS, C-gly-*Cn* H99, or C-gly-*Hc* G217B as described above, were measured in a Zeta potential analyzer (NanoBrool Omni particle, Brookhaven Instruments Corporation, Holtsville, NY) at 25°C as described (Frases et al., 2008).

### Comparative Analysis of Fungal Proteins Involved in Surface Glycan Production

Several *Cn* capsule synthesis and GXM export related proteins have been described (Chang and Kwon-Chung, 1994; Chang et al., 1996; Chang and Kwon-Chung, 1998; Chang and Kwon-Chung, 1999; Levitz et al., 2001; Janbon, 2004; Moyrand et al., 2004; Zaragoza et al., 2009; Albuquerque et al., 2012). Their respective accession numbers were recorded after searches of several databases (GenBank, Swiss-Prot/TrEMBL and Uniprot) and were grouped according to their function/families: acetyltransferases, mannosyltransferases, xylosyltransferases and miscellaneous proteins, also including proteins involved in the secretion of GXM (Albuquerque et al., 2012). Each candidate was used as a query sequence in BLAST analysis (Madera and Gough, 2002), using a threshold of 1000, an automated matrix (BLOSUM 62), and a minimum of 1000 hits. Orthologs protein hits found in *Ajellomyces capsulatus* strain Nam1/WU24 (North America 1 clade) or *A. capsulatus* strain G186AR H82/ATCC MYA-2454/RMSCC 2432 (Panama clade) were utilized to construct the **Supplementary Table 1**, which included information about protein length (number of amino acids), molecular weight, percentage of identity of *Hc* protein hits to its respective *Cn* query protein, alignment score and E-value. The family, homology and domains (and their residues) of the proteins found in the database were determined using the Pfam hidden Markov models, and the databases Interpro (http://www.ebi.ac.uk/interpro) and Pfam (http://pfam.sanger.ac.uk/). Proteins were considered related when belonging to the same family and carrying out similar metabolic processes (Finn et al., 2008; Bowden et al., 2010).

### Glycan Hydrodynamic Size Determination

Average hydrodynamic size and polydispersity values of glycan samples (1 mg/ml in PBS) were obtained by Dynamic Light Scattering (DLS) analysis in a 90Plus/BI-MAS NanoBrook Omni particle (Brookhaven Instruments Corporation, Holtsville, NY) as described (Frases et al., 2008; Guimaraes et al., 2010). The average diameter was considered the diameter of the imaginary cylinder coaxial with the thread. Size values are the average of 10 repeated measurements.

### Sera of Patients With Histoplasmosis and Cryptococcosis Patients and Healthy Individuals

Patients (>18-years old) included in the study were residents of the Rio de Janeiro State filling the proposed requirements and criteria for the classification of histoplasmosis and cryptococcosis established by the European Organization for Research and Treatment of Cancer/Invasive Fungal Infections Cooperative Group and the National Institute of Allergy and Infectious Diseases Mycoses Study Group (EORTC/MSG) Consensus Group (De Pauw et al., 2008). These comprised diagnosis confirmation by “gold standard” fungal isolation in culture from clinical specimens of probable cases; compatible clinical (X-ray imaging), epidemiological and laboratorial records, including antibody detection against histoplasmin by immunodiffusion or *Cryptococcus* antigen detection by latex agglutination (Immuno-mycologics Inc., Norman, OK, EUA). Sera obtained from 2009 to 2018 at the Imunodiagnosis branch of the Mycology Laboratory at the National Institute of Infectious Diseases (NIID), were restricted to the first serum sample collected from patients without previous treatment for any of the diseases. Control sera were obtained from healthy subjects (27-34-years old) screened for several mycosis, including histoplasmosis, cryptococcosis, paracoccidioidomycosis and aspergillosis, who offered negative results by immunodiffusion, latex agglutination and culturing. Patients <18-years old and those without clearly accessible clinical records were excluded. The use of patient sera was approved by the ethics committee of the NIID/FIOCRUZ (Protocol number 68563017.0.0000.5262).

### Indirect ELISA for Cross-Reactivity Assessment of *Hc* and *Cn* Glycans

ELISA plates were coated with 50 µl/well of a 10 µg/ml C-gly or E-gly solutions of either *Hc* or *Cn* in PBS for 1 h at 37°C, followed by an overnight incubation at 4°C. Plates were washed 3X and blocked with blocking solution (5% skin milk in TBS-T) for 1 h at 37°C. After washes, sera from healthy individuals or patients with either histoplasmosis or cryptococcosis were initially diluted at 1:100 in blocking solution. Plates were incubated at 37°C for 1 h, washed with TBS-T and incubated with goat anti-human Ig (Southern Biotech) at 1 μg/ml for 1 h at 37°C. After washes, plates were incubated with pNPP for 30 min. Absorbances were recorded at 405 nm. Experiments were performed in triplicates and results shown are the average of two independent experiments. “Cut-off value” for reactivity was defined as the average of absorbances obtained for sera of healthy individuals + 3x(standard deviation) as previously described (Guimarães et al., 2010). Histoplasmosis and cryptococcosis patient sera above the “cut-off” were considered reactive.

### Association With Macrophages

Four-to-six weeks-old female C57Bl/6 mice were obtained at the Laboratory Animal Center (NAL) of the Fluminense Federal University (Niteroi, RJ, Brazil) and housed in pathogen-free facilities, with *ad libitum* access to food and water. Their use was approved by the Ethics committee for animal use of the Fluminense Federal University (protocol 5486190618), and standard protocols followed for the isolation of bone-marrow derived macrophages (Zhang et al., 2008). Macrophages were plated at 4x10^5^ cells/well to a 24-well cell culture plate and kept in a 5% CO_2_ incubator at 37°C. *Cn* cap59 yeasts were previously incubated with 40 µg/ml of NHS-Rhodamine (ThermoScientific, USA) for 30 min at 25°C and washed extensively with excess of PBS. Cells were incubated with the distinct *Cn*-gly, *Hc*-gly or control PBS at 37°C in an orbital shaker as described above. Following incubation, cells were washed, suspended in DMEM and enumerated. Yeasts were added to the macrophages in a 5:1 (yeast: macrophage) ratio and plates incubated for 1 h/5% CO_2_. After three washes with PBS, macrophages were detached from the plates, washed and fixed overnight with a 4% formaldehyde solution. Samples were analyzed in a FACSCalibur (BD, USA), macrophages cells were gated and the percentage of fungi associated macrophages (% FL2^+^) was calculated dividing the number of fungi associated macrophages (FL2^+^ cells) over the total analyzed macrophages, as described (Cordero et al., 2016; Guimarães et al., 2019). Association experiments were performed twice, with similar results. Inhibition of interaction by each fungal glycan was calculated independently as the relative decrease of interaction levels to control *Cn cap59* for each experiment and averaged.

### Yeast Killing Assay

*Cn* Cap59 yeasts were coated with the distinct *Hc*-gly, or incubated with *Cn*-gly or PBS as positive and negative controls, respectively. Gly-coated *Cn* cap59 yeast cells were suspended in DMEM and added in a 5:1 (yeast: macrophage) ratio to 96-well culture plates containing 10^5^ macrophages/well. Plates were incubated overnight at 37°C under 5% CO_2_. The wells were washed with cold sterile PBS and macrophages lysed by adding sterile water. Aliquots were plated onto Sabouraud agar plates and incubated at room temperature for 2–3 days. The number of colony forming units was enumerated and values compared among groups.

### *Hc*-Glycans and Survival in Invertebrate Models

Larvae of *G. mellonella* (100-150 mg) in the ﬁnal instar larval stage and without any signs of dark spots or melanization and pupation were selected. To test the impact of *Hc*-gly coating on fungal virulence, 10^5^ yeasts of *Cn* cap59 were coated with 10 µg of each of the C-gly and E-gly from either *Hc* isolate or PBS as a control, as described above. Sham infection and PBS injections in the absence of yeasts (uninfected larvae) were used as controls to assess for the impact of injections and survival. A parallel survival experiment was also performed with 10^6^ yeasts coated with 100 µg of each of glycan. After washes (3X), yeasts were suspended in 10 µl of PBS and injected in the last left pro-leg of larvae of *Galleria mellonella.* At least 10 larvae were used per group in each experiment. The numbers of living larvae were monitored twice daily and recorded. Experiments were performed twice and similar results were documented.

### Statistical Analysis

All analyses were performed using GraphPad Prism version 8.00 for Windows (GraphPad Software, San Diego California USA). Ordinary One-way ANOVA test was performed for comparison among groups, with a 95% confidence interval in all experiments. Individual mean comparison to controls or between every other group was performed using Dunnett’s or Tukey’s post-tests. Survival results were analyzed by Kaplan-Meyer to determine the difference among groups (p<0.05).

## Results

### MAbs to the Capsule of *Cn* Are Reactive to *Hc* Glycans

**Figure 1:**
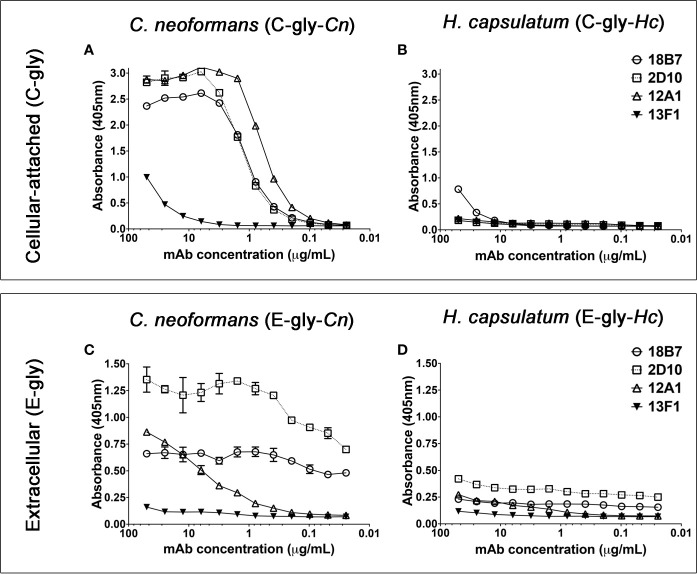
Monoclonal antibodies (MAbs) to *C. neoformans* glucuronoxylomannan (GXM) are able to bind cellular-attached (C-gly) and extracellular (E-gly) glycans of *H. capsulatum*. **(A, B)** The most widely used mAbs against CTAB-purified GXM from *C. neoformans* (12A1, 2D10, 13F1, and 18B7) were tested against DMSO extracted cellular-attached glycans of **(A)** *C. neoformans* H99 (C-gly-*Cn* H99) and **(B)** *H. capsulatum* G217B (C-gly-*Hc* G217B); the last displayed reactivity only to the 18B7 mAb, but in lower magnitude compared to control C-gly-*Cn* H99 (p<0.05). **(C, D)** GXM specific mAbs also reacted more efficiently with extracellular glycans of *C. neoformans* (E-gly-*Cn* H99) than **(D)** *H. capsulatum* (E-gly-*Hc* G217B; p<0.05), despite the similar relative reactivity ranking of the mAbs.

Serological reactivity of *H. capsulatum* (*Hc)* and *C. neoformans* (*Cn*) isolated glycans was compared using a panel of mAbs against cryptococcal GXM. Confirming previous studies (Mukherjee et al., 1993; Cordero et al., 2016), extracted C-gly-*Cn* H99 displayed a dose-dependent binding profile (**Figure 1A**) with 12A1 (IgM) displaying the highest reactivity, followed by 2D10 (IgM), 18B7 (IgG) and 13F1 (IgM). C-gly-*Hc* G217B showed only binding by the 18B7 mAb (**Figure 1B**). Binding profile of GXM mAbs to E-gly-*Hc* G217B (**Figure 1D**) was similar to that observed for E-gly-*Cn* H99 (18B7 > 2D10 > 12A1 > 13F1, **Figure 1C**), besides lower values of maximum binding. Overall, mAbs to GXM reacted to both cellular-attached C-gly-*Hc* G217B and extracellular E-gly-*Hc* G217B glycans, with the highest binding of the mAb 18B7, suggesting the presence of GXM-like epitopes in *Hc*.

### GXM mAbs React to the Yeast Phase Surface Polysaccharides of *Hc*

**Figure 2:**
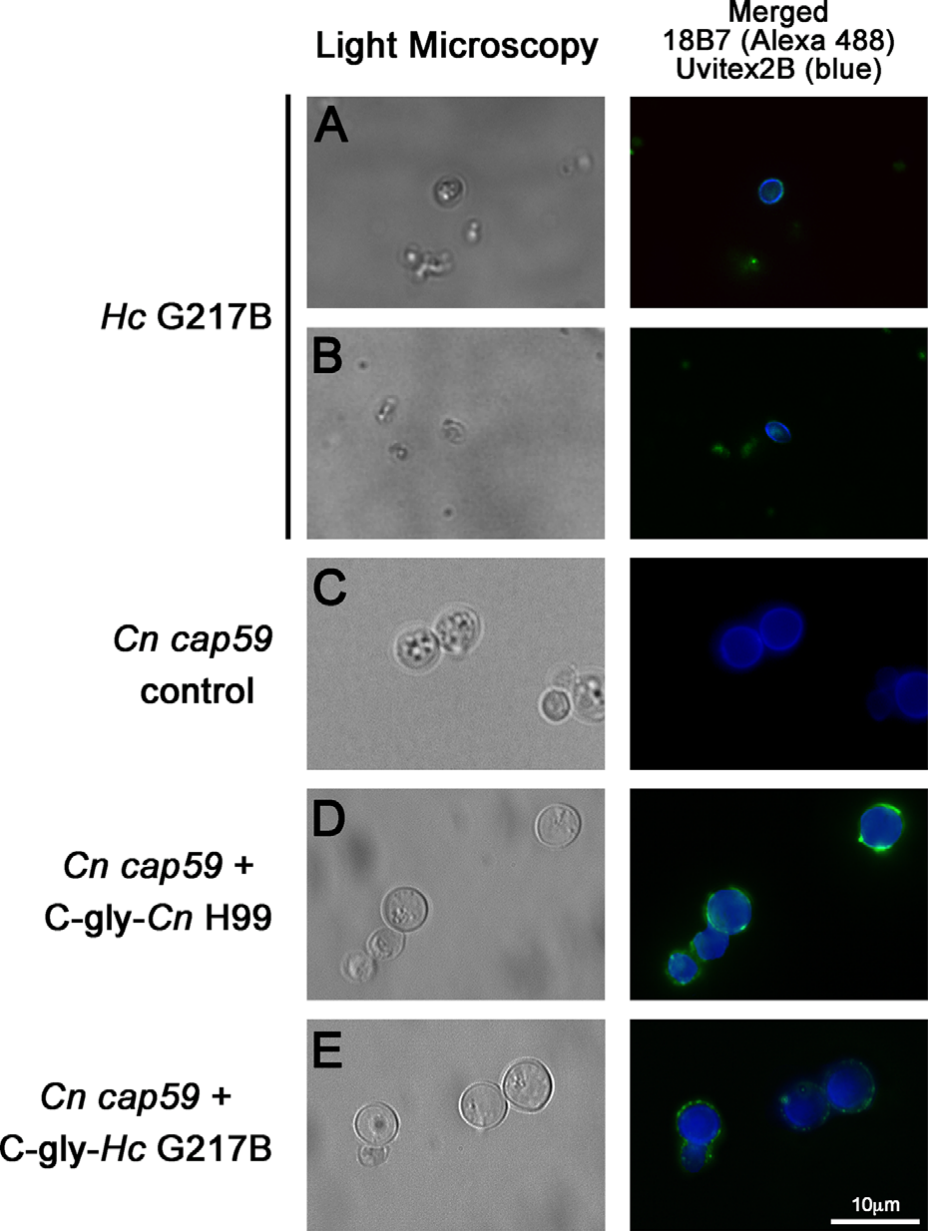
Glucuronoxylomannan (GXM) monoclonal antibodies (mAbs) bound to the C-gly on the *H. capsulatum* yeast surface, and these extracted glycans are promptly incorporated by acapsular mutants of *C. neoformans.* **(A, B)** The 18B7 mAb to *C. neoformans* GXM bound C-gly-*Hc* on the surface of the *H. capsulatum* (*Hc*) yeast, with labeling ranging from a **(A)** punctuated to a **(B)** ring pattern. **(C–E)** Acapsular *C. neoformans cap59* (*Cn cap59*) mutants were used in glycans incorporation. **(C)** *Cn cap59* incubated with PBS as negative control did not display binding by 18B7 mAb. **(D)** Incubations of *Cn* cap59 with C-gly-*Cn* H99 used as a positive control did not result in capsular structure visible by light microscopy, but surface incorporation was detected by binding of the 18B7 mAb through immunofluorescence. **(E)** *Cn* cap59 incubation with C-gly-*Hc* resulted in a similar pattern to C-gly-*Cn*, suggesting conserved anchoring mechanisms of glycans from distinct origin. Pictures were taken with an 100X objective magnification. Scale bar = 10 µm.

Cross-reactivity of GXM-mAb 18B7 to *Hc* glycans led us to evaluate the C-gly distribution and reactivity pattern over the *H. capsulatum* surface by immunofluorescence. This mAb reacts with yeast of *Cn* H99 by immunofluorescence showing a bright ring pattern (Guimaraes et al., 2010; Cordero et al., 2016). For *Hc* G217B yeast, some displayed a ring pattern, with regions showing more concentrated labeling (**Figure 2A**), although the majority of yeasts had a discrete dotted pattern of labeling along with the entire cell wall extension (**Figure 2B**).

### Incorporation of C-gly-Hc by Acapsular Mutants of *Cn*

We previously reported that *Hc* incorporates *Cn* glycans *in vitro* and *in vivo* (Cordero et al., 2016). In turn, we now examined whether *Hc* glycans could be incorporated by an acapsular mutant of *Cn* (*cap59*). The PS production by *Cn cap59* is defective and, subsequently, is not able to form a capsular network, as indicated by the absence of binding by the 18B7 mAb (**Figure 2C**). However, this strain retains the ability to incorporate exogenously added cryptococcal PS (Reese and Doering, 2003; Garcia-Rivera et al., 2004), as confirmed by C-gly-*Cn* H99 incorporation resulting in the appearance of a ring-like (e.g. circumferential) labeling, with some punctate enriched regions on *Cn cap59* yeasts after sequential incubation with mAb 18B7 (**Figure 2D**). We found that *Cn cap59* incorporates the heterologous C-gly*-Hc* G217B into its cell wall, displaying a similar pattern to the C-gly-*Cn* H99 incorporation, suggesting the presence of conserved anchoring mechanisms for glycans of distinct fungal origin (**Figure 2E**).

### *Cn* and *Hc* C-glys Analysis Reveals Distinct Glycosyl Composition and Charge

**Figure 3:**
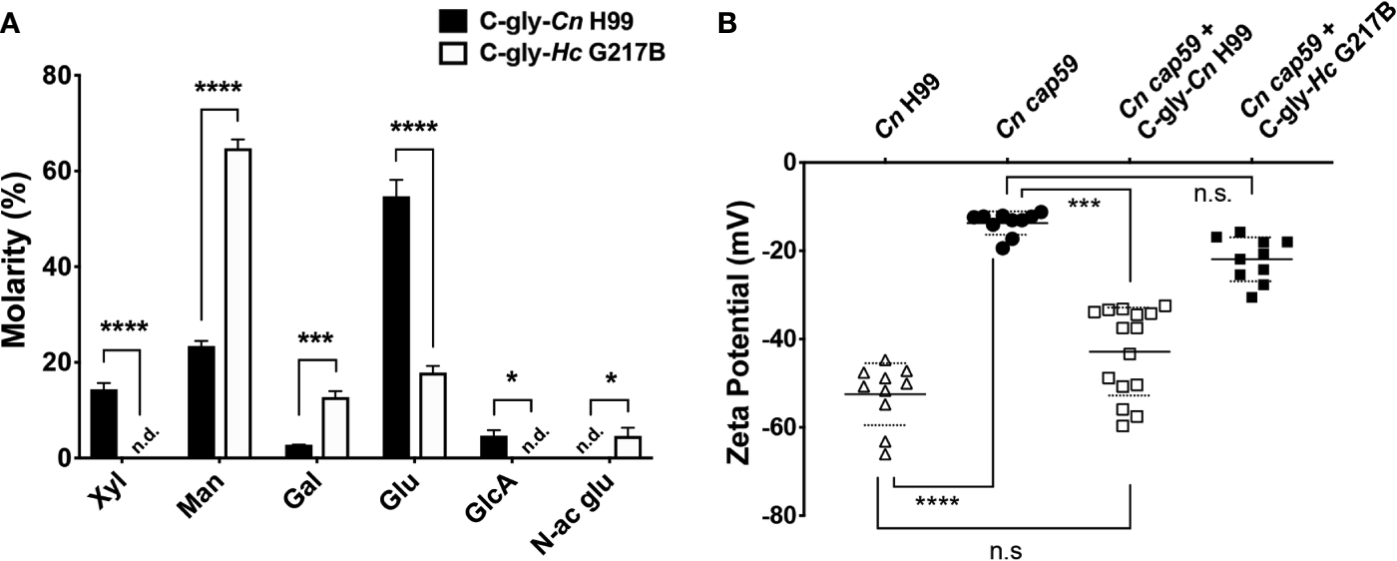
Cellular-attached glycans of *C. neoformans (Cn)* and *H. capsulatum (Hc)* had distinct glycosyl composition. **(A)** Molarity percentage of the monosaccharide blocks detected by GC-MS analysis, displaying a distinct composition with comparing the C-gly-*Cn* H99 and C-gly-*Hc* G217B. Notably, no glucuronic acid was found in C-gly-*Hc* G217B, which could influence charge. Xyl, xylose; Man, mannose; Gal, Galactose; Glu, glucose; GlcA, Glucuronic acid; N-ac glu, N-acetyl glucosamine. n.d., not detected (*p < 0.05, ***p < 0.001, and ****p < 0.0001). **(B)** Zeta potential experiments were used to measure the overall charge of cap59 *C. neoformans* yeast. Incorporation of C-gly-*Cn* H99 to *Cn cap59* significantly increased the magnitude of the negative charge, whereas the addition of C-gly-*Hc* G217B had a limited effect on the charge (***p = 0.007; ****p < 0.0001).

*Cn* and *Hc* C-gly were extracted, and their relative glycosyl composition compared (**Supplementary Figures 1A, B**). C-gly-*Cn* H99 was mostly composed of glucose (54.7%), followed by mannose (23.4%) and xylose (14.4%); glucuronic acid represented 4.7% of total composition (**Figure 3A**). C-gly-*Cn* H99 also displayed small amounts of galactose (2.8%), which may be from traces of GXMGal composing these fractions. However, C-gly-*Hc* G217B displayed a significantly different composition than C-gly-*Cn* H99 being mostly composed of mannose (64.8%; p<0.001), followed by glucose (17.9%; p<0.0001; **Figure 3A**). Galactose was also detected at 12.7% (p<0.001) and N-acetyl glucosamine in 4.6% of total carbohydrate composition, with the later being absent in C-gly-*Cn* H99 (p<0.05). As opposed to C-gly-*Cn* H99, *x*ylose and glucuronic acid were not detected in the C-gly-*Hc* G217B fractions (p<0.0001 and p<0.05, respectively).

The absence of negative charge residues of glucuronic acid in the C-gly-*Hc* G217B, led us to evaluate the overall surface charge change upon incorporation by *Cn* cap59 mutants (**Figure 3B**). *Cn* H99 controls had a zeta potential of −52.5±7.0 mV, which was much greater in magnitude than the charge of the acapsular *Cn* cap59, −13.7±2.6 mV (p<0.0001). Incorporation of C-gly-*Cn* H99 restored the values to −42.9±10.0 mV relative to *Cn* H99 (p>0.05). In contrast, C-gly-*Hc* G217B incorporation by *Cn* cap59 did not significantly affect surface charge (−21.9±5.0 mV, p>0.05).

### Cryptococcal Capsular Protein Orthologs in *Hc*

**Figure 4:**
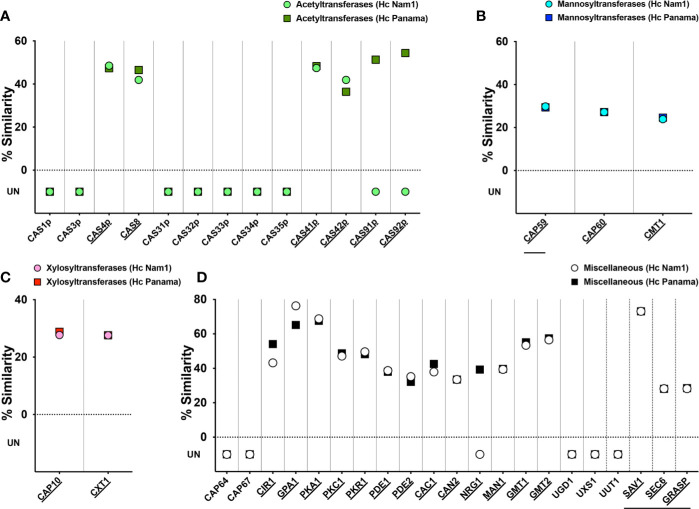
Similarity of glucuronoxylomannan synthesis and capsular production-related proteins of *C. neoformans (Cn)* and *H. capsulatum (Hc)*. *Cn* var. g*rubii* serotype A (strain H99/ATCC 208821) proteins were clustered into four groups: **(A)** acetyltransferases (green), **(B)** mannosyltransferases (blue), **(C)** xylosyltransferases (pink), and **(D)** miscellaneous (white) and sequences blasted un Uniprot in a search for orthologs from *Hc* Nam1/WU24 (light color) and the Panama/G186AR strain/H82 strains (dark colors). Similarities were recorded and used to construct the graphs of each specific protein class. Underlined protein names indicate those *C. neoformans* proteins with orthologs in *Hc*, with identical protein domains/families as annotated by Interpro/Pfam. Horizontal lines indicate proteins involved in glucuronoxylomannan (GXM) export.

Given the unexpected serological reactivity of *Hc* glycans to cryptococcal GXM-specific mAbs, we performed a high BLAST probability analysis of *Cn* capsular protein orthologs in the *Hc* genome (strains NAm1/WU24 and G186AR/H82/ATCC MYA-2454/RMSCC 2432, belonging to the NAm1 and Panama clades, respectively (Sepulveda et al., 2017) and a comparison of the relationship between the nearest orthologs allowed us to predict protein functions. A total of 39 *Cn* proteins recognized in the participation of capsule synthesis and assembly (Zaragoza et al., 2009) were organized in four main groups (acetyltransferases, mannosyltransferases, xylosyltransferases and miscellaneous) and used in a general BLAST search (**Supplementary Table 1**). In the acetyltransferase group, 4 out of 13 cryptococcal proteins (Cas4p, Cas8, Cas41p, Cas41p, and Cas42p; 31%) had orthologs in both Nam 1 and Panama strains (**Figure 4A**). Orthologs of the Cas91 and Cas92 proteins were also found specifically in the Panama strain. Regarding the three *Cn* mannosyltransferases (Cap59, Cap60, and CMT1, **Figure 4B**) and the two mannosyltransferases (Cap10 and CXT1, **Figure 4C**) all the proteins evaluated had orthologs in both *Hc* strains. Lastly, from the miscellaneous class, 15 out of 21 cryptococcal proteins (71.4%) evaluated had orthologs in both Nam1 and Panama (**Figure 4D** and **Supplementary Table 1**). Importantly, absence of orthologs to *UGD1 Cn* UDP-glucose 6 dehydrogenase, which converts UDP-glucose into UDP-glucuronic acid, nor *UUT1 Cn* UDP-glucuronic acid transporter in *Hc* genome could explain the lack of glucuronic acid in the in the C-gly-*Hc* fractions, whereas the lack of its downstream derivative xylose is directly associated to the missing ortholog to UXS1 *Cn* UDP-glucuronic acid decarboxylase, which catalyzes the conversion of UDP-glucuronic acid into UDP-xylose. Overall, from all the *Cn* proteins, 61.5% (24 out of 39) had orthologs in the *Hc* Nam1 genotype, whereas 69.2% (25/35) had orthologs in the *Hc* Panama genotype, with a high correlation of similarities to *Cn* orthologs between these two strains (**Supplementary Figure 2**). The presence of cryptococcal capsular-related proteins orthologs in the *Hc* genome is consistent with the development of similar phenotypic traits to *Cn* under certain growth conditions, particularly in presenting exposed glycan structures that are ultimately sensed and processed by host immune components.

### Cryptococcal GXM mAb 18B7 Binds Cellular-Attached and Extracellular Fractions of *Hc* From Three Distinct Clades

**Figure 5:**
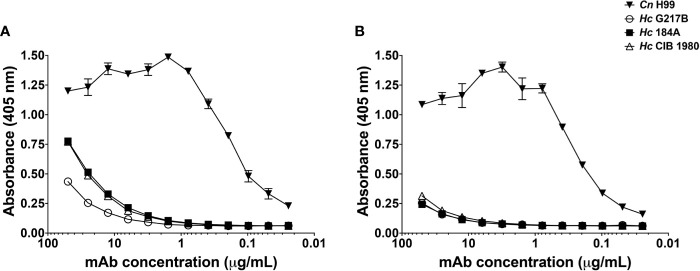
MAb 18B7 to capsular antigens of *C. neoformans (Cn)* reacted similarly to cellular-attached (C-gly) and extracellular (E-gly) polysaccharides of distinct strains of *H. capsulatum (Hc)* belonging to three different monophyletic branches. Reactivity of 18B7 mAb to **(A)** C-gly and **(B)** E-gly was compared among the *Cn* H99 control and *Hc* strains from three distinct monophyletic branches: *Hc* G217B, *Hc* G184A, and *Hc* CIB1980.

To evaluate the distribution of GXM-like epitopes on C-gly-*Hc* and E-gly-*Hc*, we have extracted these fractions from three distinct monophyletic species of *Hc* and performed an ELISA with the cross-reactive mAb 18B7 (**Figure 5**). Controls of C-gly-*Cn* H99 confirmed higher binding of mAb 18B7 in comparison to C-gly-*Hc* (**Figure 5A**). However, comparison of mAb 18B7 reactivity among C-gly-*Hc* demonstrated higher binding to C-gly-*Hc* G184A and C-gly-*Hc* CIB1980, and slightly less binding to C-gly-*Hc* G217B from the reference strain. Evaluation of mAb 18B7 binding to E-gly also revealed the highest binding to E-gly-*Cn* H99 as expected (**Figure 5B**). All three E-gly-*Hc* evaluated (*Hc* G217B, *Hc* G184A, and *Hc* CIB1980), however, displayed similar low reactivity to mAb 18B7 (p>0.05).

### Size of *Hc* Glycans From Three Distinct Clades

**Figure 6:**
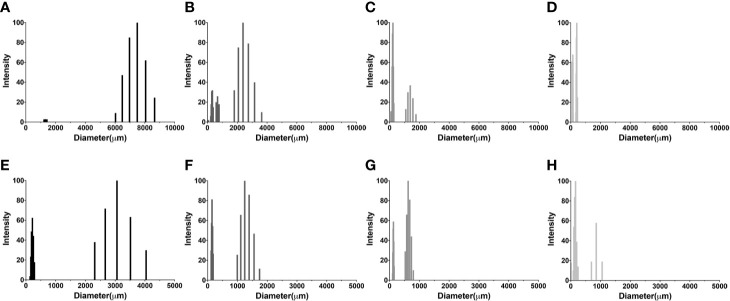
Comparison of C-gly and E-gly dimensions of *C. neoformans (Cn)* and strains of *H. capsulatum (Hc)* from three different monophyletic branches (Nam 2 *Hc* G217B, Panama *Hc* G186, and LAm CIB 1980) reveals structural differences and distinct architecture. Dynamic light Scattering (DLS) was used to measure the fiber dimensions of cellular-attached and extracellular glucans of both fungi. Cellular-attached glycans of: **(A)** C-gly-*Cn* H99, **(B)** C-gly-*Hc* of *Hc* G217B, **(C)** C-gly-*Hc* of *Hc* G184A and **(D)** C-gly-*Hc* of Hc CIB1980 strains. Extracellular glycans of: **(E)** E-gly-*Cn* H99, **(F)** E-gly-*Hc* of *Hc* G217B, **(G)** E-gly-*Hc* of *Hc* G184A, and **(H)** E-gly-*Hc* of *Hc* CIB1980 strains.

The slight differences in reactivity of *Cn* GXM mAb to C-gly-*Hc and* E-gly-*Hc* led us to examine the *Hc* surface glycans in more refined detail. We have compared the average hydrodynamic sizes of cellular-attached glycans (C-gly) isolated from *Cn* H99 (C-gly-*Cn* H99) and the three distinct isolates of *Hc* (C-gly-*Hc*) yeast cells (**Figure 6**). As our reference, C-gly-*Cn* H99 exhibited two main populations, with a small group ranging from 1,230 to 1,421 nm and a larger fraction from 6030 to 8654 nm (effective diameter= 7,212 nm, **Figure 6A**). The C-gly-*Hc* diameter had slight variations depending on the clade the strain belonged to. C-gly-*Hc* G217B displayed a small population ranging from 72 to 778 nm and a larger population from 1,800 to 3,600 nm (effective diameter= 1,802 nm, **Figure 6B**). The C-gly*-Hc* G184A in turn, overall displayed two populations of smaller sizes than the C-gly-*Hc* G217B, with a small population ranging from 83 to 282 nm and a larger population from 1,085 to 1,770 nm (effective diameter 487 nm, **Figure 6C**). The C-gly*-Hc* CIB 1980 displayed the shortest fibers, with a small population ranging from 129 to 165 nm and a larger population from 350 to 450 nm (effective diameter 254 nm, **Figure 6D**).

Regarding the extracellular glycans, the E-gly-*Cn* control displayed two populations, a small from 139 to 286 nm and a larger ranging from 2,312 to 4,040 nm (effective diameter= 1,930 nm, **Figure 6E**). *Hc* yeasts secreted smaller fibers, with the E-gly-*Hc* G217B displaying a small population from 118 to 185 nm and a larger from 995 to 1743 nm (effective diameter= 808 nm, **Figure 6F**). The E-gly-*Hc* G184A displayed a small from 101 to 152 nm and a larger from 528 to 799 nm (effective diameter= 445 nm, **Figure 6G**). In the other hand, the E-gly-*Hc* CIB 1980 displayed the largest fibers with a small population from 5 to 244 nm and a larger from 694 to 1,054 nm (effective diameter of 290 nm, **Figure 6H**). Overall, the small differences observed for the C-gly-*Hc* were also similarly observed for the E-gly-*Hc* (*Hc* G217B> *Hc* G184A > *Hc* CIB 1980) from distinct strains.

### Serological Cross-Reactivity of Sera From Cryptococcosis Patients to *Hc* Glycans

**Figure 7:**
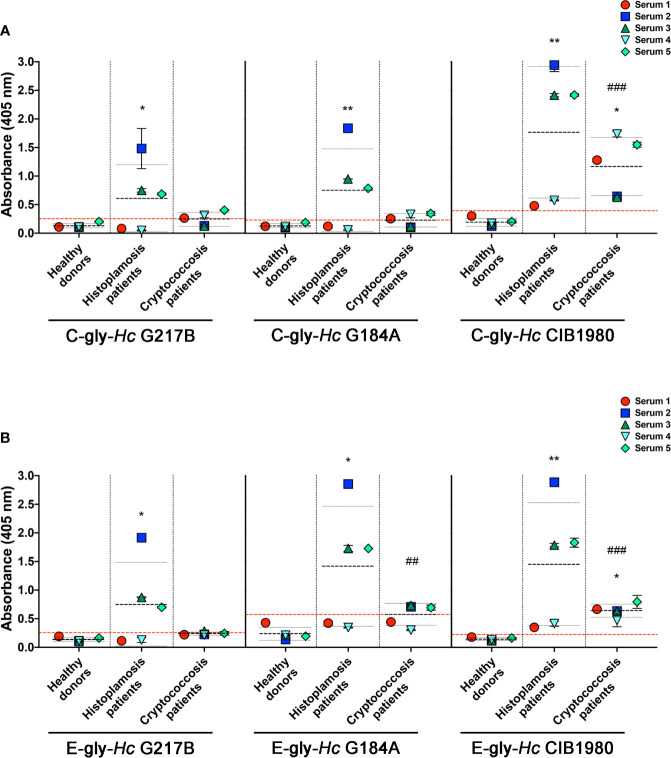
Serological cross-reactivity of sera from patients with cryptococcosis to *H. capsulatum* cellular-attached (C-gly-*Hc*) and extracellular (E-gly-*Hc*) polysaccharides from strains from three distinct monophyletic groups, *Hc* glycans were attached to ELISA plate and the reactivity of sera from patients with cryptococcosis against *Hc*-gly was compared to reactivity of negative control sera from heathy subjects and controls of sera from patients with histoplasmosis. **(A)** Reactivity of the cellular-attached glycans C-gly *Hc* G217B, C-gly *Hc* G184A, and C-gly C-gly-*Hc* of *Hc* CIB1980 strains. **(B)** Reactivity of the Extracellular glycans E-gly-*Hc* of *Hc* G217B, E-gly-*Hc* of *Hc* G184A, and E-gly-*Hc* of *Hc* CIB1980 strains. *P ≤ 0.05 and **p ≤ 0.01; comparison of histoplasmosis or cryptococcosis patient’s sera reactivity to *Hc* glycans versus healthy subjects control; ^##^p ≤ 0.01, ^###^p ≤ 0.001; comparison among the distinct Hc glycans demonstrated higher reactivity of cryptococcosis patient’s sera to *Hc* CIB 1980 glycans versus the respective glycan from either *Hc* G217B or *Hc* G184A.

**Figure 8:**
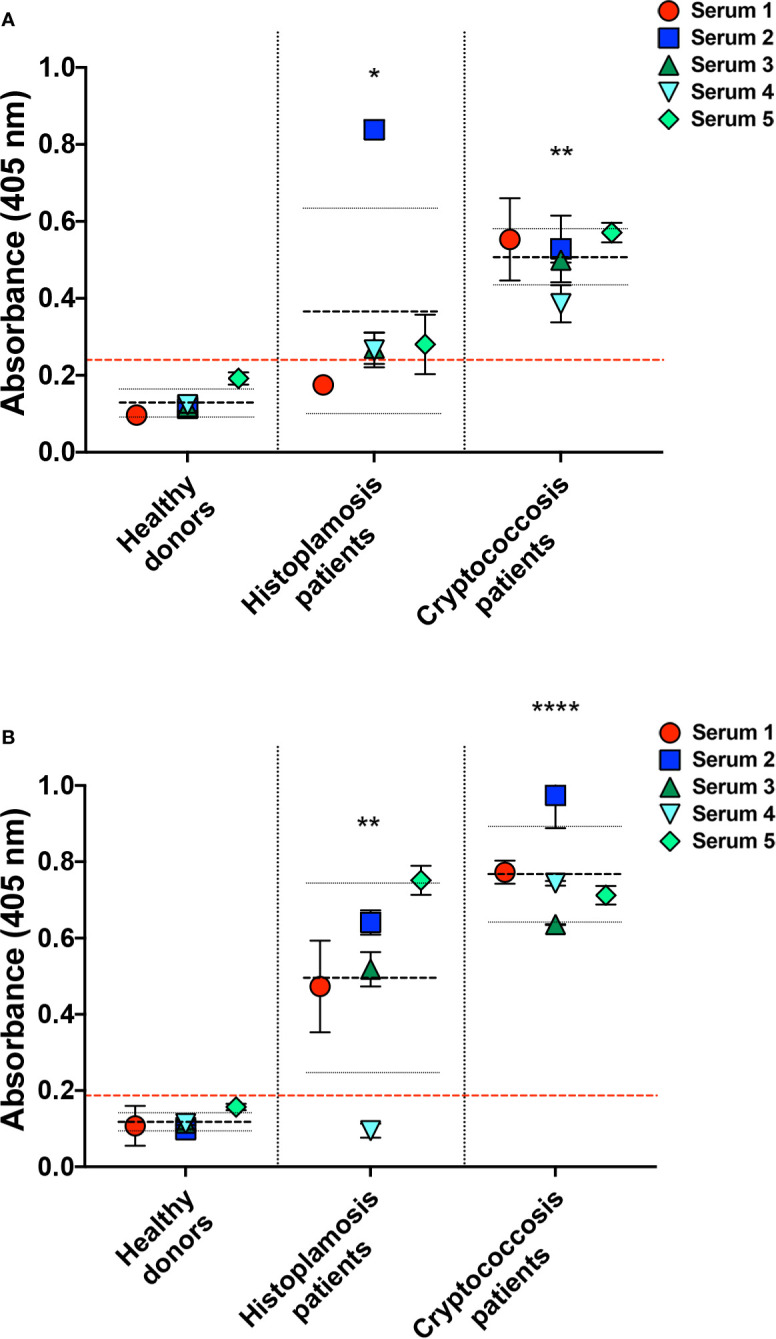
Serological cross-reactivity of sera from patients with histoplasmosis to *C. neoformans* capsular (C-gly-*Cn*) and extracellular (E-gly-*Cn*) polysaccharides confirmed the presence of immunogenic epitopes in *Hc* glycans. Reactivity of antibodies from histoplasmosis patient sera was evaluated against *C. neoformans (Cn)* glycans **(A)** C-gly-*Cn* H99 and **(B)** E-gly-*Cn* H99, confirming the presence of antibodies raised by *Hc* glycans that are able to recognize similar epitopes in *Cn* glycans. As a controls, reactivity was compared to negative control sera from healthy individuals and positive control sera of cryptococcosis. *P ≤ 0.05, **p ≤ 0.01, ***p ≤ 0.0001; comparison of histoplasmosis or cryptococcosis patient’s sera reactivity to *Cn* glycans versus healthy subjects control.

The reactivity of 18B7 mAb to *Hc-*gly led us to evaluate whether antibodies naturally generated during cryptococcosis that react with *Cn*-gly were also able to bind to *Hc*-gly. Sera of five patients with cryptococcosis were initially screened against C-gly*-Cn* H99 and E-gly*-Cn* H99 to confirm the presence of reacting antibodies to these fractions (as controls for serum from patients with cryptococcosis and their reactivity, shown in **Figures 8A, B**, respectively). Then, their reactivity against C-gly*-Hc* and E-gly*-Hc* of distinct clades was compared to sera from patients with histoplasmosis. Overall, average absorbances for sera from patients with cryptococcosis reacting to either C-gly-*Hc* G217B or C-gly-*Hc* G184A had similar values to “cut off”, as 3 out of 5 sera from cryptococcosis patients (sera 1, 4 and 5) displayed good reactivity to both (**Figure 7A**). When sera from cryptococcosis patients were tested against C-gly-*Hc* CIB1980, average absorbances were about 3 times higher than “cut off” values, with all five sera demonstrating good reactivity (*p<0.05). Therefore, reactivity comparison of sera from cryptococcosis patients among the three C-gly-*Hc* indeed revealed that the best reactivity was to C-gly-*Hc* CIB1980 (###p<0.001).

Regarding the reactivity of sera from cryptococcosis patients to E-gly-*Hc*, only one serum (serum 2) had good reactivity to E-gly-*Hc* G217B, with average values below the “cut-off” (**Figure 7B**). Reactivity to E-gly-*Hc* G184A was observed with three out of five sera (sera 2, 3, and 5), with average of absorbance above the “cut off”. Lastly, all sera from cryptococcosis patients displayed reactivity to E-gly-*Hc* CIB1980, with average of absorbances about three times than “cut off” (*p<0.05), configuring the best reactivity among the E-gly-*Hc* (###p<0.001).

### Cross-Reactivity of Antibodies From Histoplasmosis Patients to *Cn*-Glycans

To also evaluate the humoral immunogenicity of *Hc*-gly, we tested the cross-reactivity of sera from patients with histoplasmosis against *Hc*-gly, and verified whether raised antibodies also recognized epitopes in *Cn*-gly (**Figure 8**). Their reactivity to C-gly-*Hc* and E-gly*-Hc* from distinct *Hc* strains are found on **Figures 7A, B**, respectively. These sera displayed average absorbances for C-gly-*Hc* G217B and C-gly-*Hc* G184A of 2.4 and 3.2 times higher than “cut-off” values (*p<0.05 and **p<0.01, respectively; **Figure 7A**), with three out of five sera demonstrating reactivity (sera 2, 3, and 4). However, average absorbance was 4.5 times higher to C-gly-*Hc* CIB1980 (**p<0.01; **Figure 7A**), with all sera reacting against this fraction, demonstrating best reactivity among the C-gly-*Hc* (###p<0.001). Reactivity to E-gly-*Hc* of distinct origin followed a similar behavior, but with fairly higher values of absorbances (**Figure 7B**).

Antibodies raised against *Hc*-gly and present in the sera of patients with histoplasmosis also cross-reacted with epitopes present in *Cn*-gly. Average absorbance for sera from patients with histoplasmosis was 1.5 higher than “cut-off” values (*p<0.05), with 4 out of 5 sera (sera 2,3,4 and 5) reacting to C-gly-*Cn* H99 (**Figure 8A**). Regarding reactivity to E-gly-*Cn* H99 average absorbance was three times higher than “cut-off” (**p<0.01), as 4 out of 5 sera (sera 1, 2, 3, and 5) demonstrating high reactivity (**Figure 8B**). Overall, cross-reacting antibodies in sera from patients with histoplasmosis better recognized epitopes present in E-gly-*Cn* H99 fractions,

### *H. capsulatum* Glycans Inhibited Phagocytosis and Antifungal Activity by Peritoneal Macrophages.

**Figure 9:**
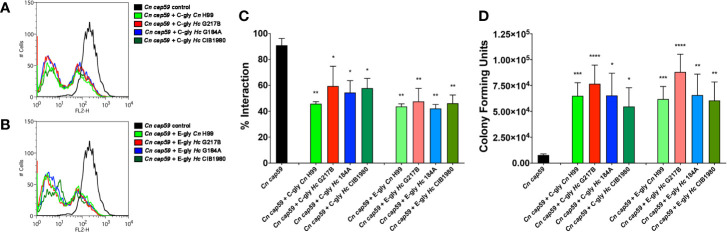
*H. capsulatum* glycans (*Hc*-gly) incorporation by a cap59 acapsular mutant of *C. neoformans (Cn)* confers resistance to phagocytosis by macrophages. **(A, B)** Representative histograms demonstrating the interactions of uncoated or coated *Cn cap59* with **(A)** C-gly-*Hc* or **(B)** E-gly-*Hc* from *H. capsulatum (Hc)* strains from distinct monophyletic branches. **(C)** Incorporation of either C-gly-*Hc* or E-gly-*Hc* by the acapsular mutant *Cn cap59* significantly inhibited the fungal association with murine peritoneal macrophages, as values similar to *Cn* gly controls**. (D)** Co-culture of macrophages with *Cn cap59* coated with C-gly-*Hc* or E-gly-*Hc* from distinct strains also inhibited the killing of yeasts by murine peritoneal macrophages. Bars represent mean ± standard error of quadruplicates. *P ≤ 0.05; **p ≤ 0.01; ***p ≤ 0.001; ****p ≤ 0.0001.

The antiphagocytic properties of C-gly and E-gly from *Hc* were evaluated and compared to the established antiphagocytic C-gly-*Cn* and E-gly-*Cn* (**Figures 9A, B**). Relative to untreated yeasts, *Cn cap59* yeasts coated with C-gly from the distinct *Hc* strains equally displayed enhanced resistance to phagocytosis by peritoneal macrophages (*Hc* G217B, 41% inhibition; *Hc* G184A, 46% and *Hc* CIB 1980, 42%), which were at levels similar to that achieved following incubation of *Cn cap59* with C-gly-*Cn* H99 (54% inhibition; **Figures 9A, C**). E-gly-*Hc* also inhibited phagocytosis of *Cn cap 59* coated yeasts (*Hc* G217B, 52% inhibition; *Hc* G184A, 56%, and *Hc* CIB 1980, 54%), similarly to E-gly-*Cn* (56% inhibition; **Figures 9B, C**). Additionally, resistance to killing by macrophages was also enhanced when cap59 *C. neoformans* were coated with either C-gly or E-gly from the three *Hc* strains (**Figure 9D**).

### *Hc* Glycans Enhance the Virulence of Acapsular Mutants of *Cn* in Invertebrate Models of *Galleria mellonella*

**Figure 10:**
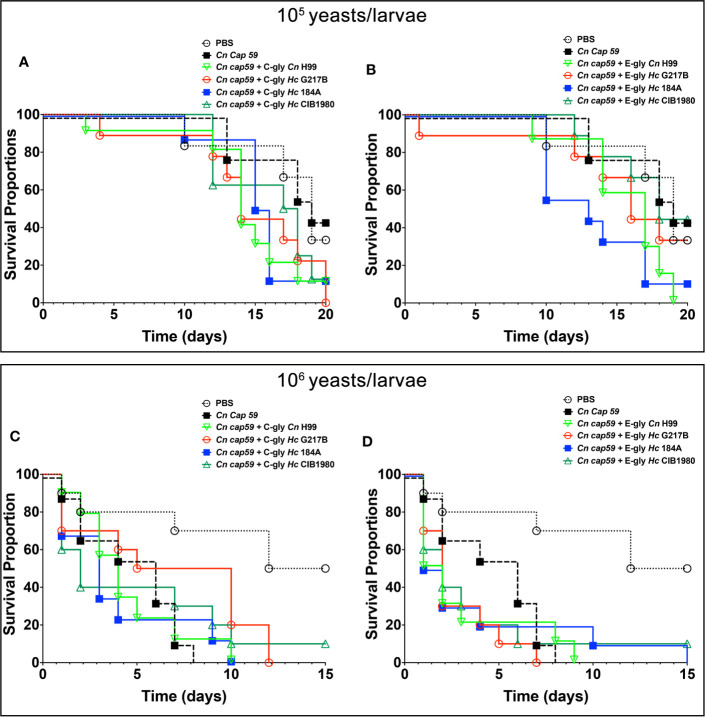
*H. capsulatum* glycans (*Hc*-gly) are able to turn the avirulent *C. neoformans (Cn) cap59* into virulent yeasts. **(A, B)** *Cn cap59* were coated with **(A)** C-gly or **(B)** E-gly from distinct strains of *Hc* or controls yeast *Cn* H99 and used to infect (10^5^ yeast/larvae) of *Galleria mellonela*. **(C, D)** *Cn cap59* were coated with **(C)** C-gly or **(D)** E-gly from distinct strains of *Hc* or controls yeast *Cn* H99 and 10^6^ yeast/larvae used for infections.

We used *G. mellonella* larvae as a model to investigate the impact of *Hc* glycans in fungal virulence. *Cn cap59* yeasts were coated with *Hc* glycans and used to infect the larvae, Infections with uncoated *Cn cap59* or coated with *Cn* H99 glycans were used as controls. As expected, controls of C-gly*-Cn* H99 and E-gly-*Cn* H99 coated *Cn* cap59 infected larvae of *G. mellonella* died faster than those infected with uncoated *Cn cap59* control (p<0.05). From the groups of *Hc*-glycans coated *Cn cap59*, only the C-gly-*Hc* G217B coated *Cn cap59* killed the larvae faster than uncoated *Cn cap59* control (p=0.035; **Figure 10A**), whereas the C-gly-*Hc* G184A displayed a trend for higher killing capacity (p=0.076). From the E-gly-*Hc* coated *Cn* cap59 yeasts, only those coated with E-gly-*Hc* G184A were able to reduce larvae survival (p<0.05; **Figure 10B**).

Increasing the inoculum (10^6^ yeasts/larvae) resulted in faster killing of the larvae by *Cn* cap59 controls and no difference was observed among groups (**Figures 10C, D**), despite of a trend for accelerated death with the E-gly-*Hc* G217B coated *Cn cap59* infected larvae in comparison to *Cn cap59* control (p=0.067).

## Discussion

Polysaccharides compose up to 80% of the fungal cell wall (Erwig and Gow, 2016; Gow et al., 2017), displaying several functions related to immune recognition and playing a central role in fungal pathogenesis (Gow et al., 2017). The fungal cell wall is a dynamic and metamorphic structure. In *Candida albicans*, for example, adaptation to environmental stress, including changes in carbon sources, involves a complex regulatory network by switching its metabolism and morphogenesis, including cell wall remodeling and, altogether, alterations in the cell wall result in changes in virulence (Brown et al., 2014). Simpler direct mechanisms also are involved in the regulation of the cell wall thickness and composition, such as the secretion of Eng1 β-glucanase by *H. capsulatum* (*Hc*) (Garfoot et al., 2016), which trims the β-1,3-glucans off the cell wall, reducing its exposure and subsequent immune recognition through Dectin-1 to contribute to the immune escape and enhanced the virulence of this fungus (Brown, 2016).

*Ascomycete* dimorphic fungi, such as *Hc* (Klimpel and Goldman, 1988), *P. brasiliensis* (San-Blas and Vernet, 1977; Tomazett et al., 2005) and *Blastomyces dermatitidis* (Hogan and Klein, 1994) are able to modify the recognition of β-1,3-glucan by innate immune cells by altering the production and display of α-1,3-glucan during morphogenic transformation from hyphal forms to yeast cells. In contrast, loss of this polysaccharide is linked to a reduction of virulence *in vivo* (San-Blas and Vernet, 1977; Rappleye et al., 2004).

In *C. neoformans* (*Cn*), α-1,3-glucan is responsible for GXM fibers attachment to the cell surface, as the absence of this polysaccharide results in acapsular phenotypes despite normal GXM shedding mechanisms (Reese and Doering, 2003; Reese et al., 2007). Previous observations by Reese and Doering demonstrated that α-1,3-glucan expressing *Hc* is also able to anchor cryptococcal GXM and form a capsule-like structure (Reese and Doering, 2003). Further observations by our group (Cordero et al., 2016) mechanistically demonstrated that cryptococcal GXM incorporation by *Hc* had implications on biofilm formation and fungal resistance to phagocytosis, resulting in enhanced fungal virulence and worst prognosis of the co-infection. However, the fact that *Hc* incorporated cryptococcal GXM and cross-reactivity of some mAbs generated against cryptococcal GXM to *Hc* filamentous and yeast cell surface, suggested the production and expression of GXM-like fibers by this fungus.

To address this hypothesis, in the present study, we carried out the characterization of cellular-attached and secreted extracellular pools of glycans (C-gly and E-gly, respectively) obtained from *Hc* and initially tested their serological reactivity against a panel of mAbs to *Cn* GXM. We decided to keep the term glycans as a general denomination for fibers composed by glycosidic-bound monosaccharides, as no structural determination was carried out. C-gly and E-gly extracted from the reference *Hc* G217B strain reacted only with the 18B7 mAb, but at lower levels when compared to *Cn* fractions. Lower affinity of *Hc* glycans to GXM antibodies might be dictated by structural differences and/or relative abundance of the target epitopes. It is well documented that the mAb 13F1 differs in specificity to others in the panel, and usually labels the *Cn* yeast in a punctate pattern throughout the capsule (Nussbaum et al., 1997; Cleare et al., 1999). This mAb also shows a discrete reactivity to *P. brasiliensis* C-gly by ELISA, and a punctuated labeling pattern on the yeast surface by immunofluorescence (Albuquerque et al., 2012). Nevertheless, it is clear that the absence of reactivity of the 13F1 mAb to either *Hc* C-gly and E-gly indicates that its target epitope might be absent in this glycan pool.

The reactivity of the 18B7 mAb, which is by far the most used in studies of cryptococcal capsule characterization, to *Hc* glycans by ELISA led us to evaluate its binding profile and target epitope distribution on *Hc*. MAb 18B7 labeling ranged from a dotted to a ring pattern, confirming previous indications by our group (Cordero et al., 2016). This same binding pattern was originally reported for *Cn* (Casadevall et al., 1998) and other GXM-like polysaccharide producing fungi, such as *C. liquefaciens* (Araujo et al., 2017), *T. asahii* (Fonseca et al., 2009), *T. mucoides* (Zimbres et al., 2018) and *P. brasiliensis* (Albuquerque et al., 2012).

As a GXM-like component, C-gly-*Hc* could be promptly incorporated by the *Cn cap59* acapsular mutant, forming a capsule-like structure, with some dotted regions, resembling the capsule formed when C-gly-*Cn* was used. A similar profile was also observed for the incorporation of other GXM-like components of the most diverse origin, likely indicating a shared property among them (Fonseca et al., 2009; Albuquerque et al., 2012; Araujo et al., 2017; Zimbres et al., 2018). As for *Cn* GXM, C-gly-*Hc* incorporation could occur *via* α-1,3-glucan (Reese and Doering, 2003) or attachment to cell wall chitin (Ramos et al., 2012).

An intriguing point is the structural characterization of these GXM-like molecules. For *Cn*, GXM is composed of a backbone of mannan with substitutions of xylose and glucuronic acid, creating a high diversity of motifs and possible combinations (Cherniak et al., 1998; McFadden et al., 2006). *T. asahii* GXM-like, in turn, displays a relatively higher number of mannosyl units and distinct positions of xylosyl substitutions per motif (Fonseca et al., 2009). The closely related *T. mucoides* displays a very similar composition to *T. asahii*, except for the higher number of glucuronic acid substitutions (Zimbres et al., 2018). The *P. brasiliensis* GXM-like polysaccharide is mainly composed of mannose and galactose, and traces of glucose, xylose, and rhamnose, with an absence of glucuronic acid (Albuquerque et al., 2012). Similarly, in surface glycans of *Hc*, mannose and glucose were detected, in addition to small amounts of galactose and N-acetyl glucosamine; however, the main observation in our analyses was the absence of xylose and glucuronic acid. However, we cannot rule out the possibility that these identified *Hc* glycans could have similar compositions to polysaccharides antigens previously described (Zancope-Oliveira et al., 1994), or to the cross-reacting galactomannans of *P. brasiliensis* (San-Blas and San-Blas, 1982).

As expected, due to the absence of both residues, incorporation of C-gly-*Hc* G217 by *Cn cap59* had no effect on surface charge. In contrast, the incorporation of C-gly-*Cn* H99 bearing glucuronic acid and xylose clearly resulted in a more negative charge, similar to *Cn* H99 controls. Xylose seems not to alter the overall charge of polysaccharide fibers in a wide range of pH (Barbosa et al., 2019), including physiological pH 7.2 used in our experiments, Therefore, pKa of glucuronic acid in the range of ~3.0 confers a more negative charge to GXM.

*Cn* mutants deficient in the production of glucuronic acid and xylose have been described in the literature both with defective capsule production. A *Cn* mutant deficient in UDP-glucose 6 dehydrogenase (*UGD1*), lacks UDP-glucuronic acid and its downstream product UDP-xylose, displaying an acapsular avirulent phenotype and are highly sensitive to temperature and environmental stress (Moyrand et al., 2002; Moyrand and Janbon, 2004; Griffith et al., 2004). A mutant deficient in UDP-xylose synthase (*UXS1* or also called UDP-glucuronic acid decarboxylase), lacks UDP-xylose and displays a hypocapsular, hypovirulent phenotype, and are not recognized by some mAbs to GXM (Moyrand et al., 2002; Griffith et al., 2004). Intriguingly, these mutants displayed accumulated UDP-glucuronic acid to levels 64 times higher than WT *Cn*.

A comparative protein database search for the presence of homologous proteins in two distinct genotypes of *Hc* (NAm1/WU24 and G186AR/H82) to those involved in the GXM synthesis and capsule architecture assembly in *Cn*, also revealed the absence of *UUT*1, *UGD1* and *UXS1*, orthologs in both *Hc* genomes, in agreement with the lack of these residues in C-gly-*Hc* and supporting the aforementioned glycosyl composition results. Overall, these would effectively correlate to small fibers observed for C-gly-*Hc* and capsule absence on *Hc* yeasts, as opposed to C-gly-*Cn*, cationic bridges with glucuronic acid residues result in the formation of larger fiber composing the cryptococcal capsular network (Nimrichter et al., 2007).

However, the suggestive presence of GXM-like molecules on the surface of *Hc* led us to perform additional searches that included acetyltransferases, mannosyltransferases, xylosyltransferases and miscellaneous (Chang and Kwon-Chung, 1994; Chang et al., 1996; Chang and Kwon-Chung, 1998; Chang and Kwon-Chung, 1999; Levitz et al., 2001; Janbon, 2004; Moyrand et al., 2004; Zaragoza et al., 2009; Albuquerque et al., 2012). BLAST displayed high similarity to *Cn* and *Hc* proteins, indicating both species share metabolic pathways required for the synthesis of molecules that resemble *Cn* GXM, and a possible evolutionary relationship between these two species. All the *Cn* protein groups evaluated had orthologs in *Hc*, supporting the presence of a GXM-like structure in the last. In addition, it must be stressed that one important gene, cap67 that encodes a cryptococcal chitin deacetylase, had no orthologs in neither *Hc* strains evaluated. The absence of this gene in *Cryptococcus* sp. is related to a capsular deficiency phenotype, despite a regular secretion of GXM (Jacobson et al., 1982; Reiss et al., 1986); however, *Hc* might express other genes that could function similarly and compensate for the absence of these orthologs.

Supporting these results, the 18B7 mAb raised against cryptococcal GXM reacted with C-gly and E-gly of three distinct strains of *Hc*, suggesting the presence of similar GXM epitopes across distinct clades of this fungus. Slight differences in reactivity among strains might be explained by differences in molecular dimensions and epitope diversity (Nimrichter et al., 2007; Albuquerque et al., 2014). On the other hand, distinct reactivity by ELISA using sera of cryptococcosis patients might also suggest that these C-gly and E-gly from distinct *Hc* strains might have a distinct set of epitopes, also found in cryptococcal GXM, with best cross-reactivity to glycans of *Hc* CIB1980. Further evaluation by ELISA also demonstrated that serum of histoplasmosis patients also have antibodies able to recognize cryptococcal C-gly and E-gly, suggesting once more a similarity between these two species and that the similar epitopes found in *Hc* are sufficiently immunogenic to induce a measurable humoral response.

Regardless of the relative composition of the GXM-like polysaccharides and the serological reactivity to *Cn* GMX mAbs, C-gly-*Hc* and E-gly-*Hc* were effectively incorporated onto the surface of *Cn* cap59 acapsular mutants impaired phagocytosis by macrophages and enhancing yeast intracellular survival in these phagocytes. These results provide additional evidence for the similarity among the GXM-like polysaccharides and further support their protective activity to other fungi against phagocytes, suggesting that they might be involved in fungal pathogenesis in distinct models (Fonseca et al., 2009; Albuquerque et al., 2012; Araujo et al., 2017; Zimbres et al., 2018).

Overall, together with previous studies showing incorporation of *Cn* GXM by *Hc* and virulence enhancement *in vitro* and *in vivo* (Cordero et al., 2016), here we also show the capacity of *Hc* to produce a GXM-like molecule. Therefore, the presence of the GXM-like polysaccharides across the fungal kingdom, in both Ascomycetes and Basidiomycetes, and their role as a crucial component for the pathogenesis process as well as their capacity for eliciting humoral responses offers additional support that they could be targeted for the treatment of mycosis. As such, certain distinct scenarios could be developed to improve the portfolio of strategies: (i) drug design of new antifungal molecules that block the pathways involved in the GXM synthesis, which would have a wide antifungal spectrum and (ii) the use of cross-reactive mAbs, as for example, the 18B7 mAb, which offered protection in infection models of *Cn*, in further passive immunization studies involving other models or for use in pan-fungal radioimmunotherapy (Nosanchuk and Dadachova, 2011), and (iii) targeted modulation of the immune response to polysaccharides to speed the resolution of infection and benefit the host. These examples focused on approaches to surface GXM-like compounds provide a strong foundation for a very promising path forward to the design of new antifungal strategies (Rappleye et al., 2007; Amarsaikhan and Templeton, 2015).

## Data Availability Statement

The raw data supporting the conclusions of this article will be made available by the authors, without undue reservation.

## Ethics Statement

The animal study was reviewed and approved by animal institute committee of the Fluminense Federal University (protocol 5486190618).

## Author Contributions

All authors contributed to conception and design of the study. DG, CR, MF, LH, GA, RC, and AG performed the experiments. DG, CN, MF, RC and AG organized the database. DG, CN, MF and AG performed the statistical analysis. All authors designed experiments and participated in scientific discussions. DG, CN, MF, JN, RC and AG wrote the first draft of the manuscript. All authors contributed to the article and approved the submitted version.

## Funding

AG was supported by grants from the Brazilian agencies Conselho Nacional de Desenvolvimento Científico e Tecnológico (CNPq, grants 311470/2018-1) and Fundação Carlos Chagas de Amparo à Pesquisa no Estado do Rio de Janeiro (E-26/202.696/2018).

## Conflict of Interest

The authors declare that the research was conducted in the absence of any commercial or financial relationships that could be construed as a potential conflict of interest.

## Supplementary Material

Supplementary Figure 1 Chromatograms of the glycosyl composition of *C. neoformans* and *H. capsulatum* C-glycans. **(A)** C-gly-*Cn* H99 and **(B)** C-gly-*Hc* G217B displayed a distinct composition.

Supplementary Figure 2 Correlation the *H. capsulatum* Nam1/WU24 and the Panama/G186AR strain/H82 strain regarding their similarities to the *C neoformans* var. grubii serotype A (strain H99/ATCC 208821) strain. Circle filling colors denote the acetyltransferases (green), mannosyltransferases (blue), acetyltransferases (pink), and miscellaneous (white) groups. Similarity values of proteins from the two strains displayed a correlation (R2 = 0.70, ****p<0.0001; UN – unidentified in the database).

Supplementary Table 1 Glucuronoxylomannan synthesis and capsular production-related proteins of *C. neoformans* and their respective orthologs in *H. capsulatum* Nam1/WU24 and the Panama/G186AR strain/H82 strains. Cryptococcal proteins were classified as acatyltransferases, mannosyltransferases, xylosyltransferases, and miscellaneous, including polysaccharide transport and GXM export proteins. Underlined protein names indicate those *C. neoformans* proteins with orthologs in *H. capsulatum*, with identical protein domains/families as annotated by Interpro/Pfam.
