# Supplementary figures and images for: Histoplasma capsulatum Glycans From Distinct Genotypes Share Structural and Serological Similarities to Cryptococcus neoformans Glucuronoxylomannan
Source: Front Cell Infect Microbiol. 2021 Jan 8;10:565571. doi: 10.3389/fcimb.2020.565571 (PMC7874066; doi:10.3389/fcimb.2020.565571)

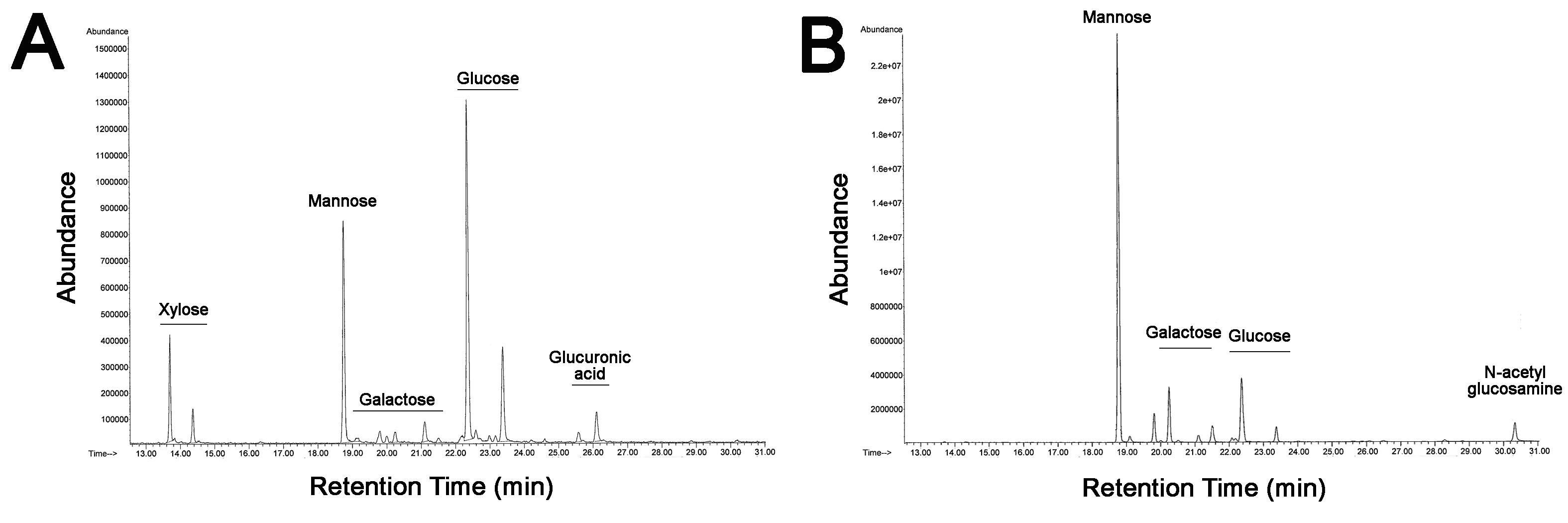

Supplement: Supplementary Figure 1 — Chromatograms of the glycosyl composition of C. neoformans and H. capsulatum C-glycans. (A) C-gly-Cn H99 and (B) C-gly-Hc G217B displayed a distinct composition. [file Image_1.tif]

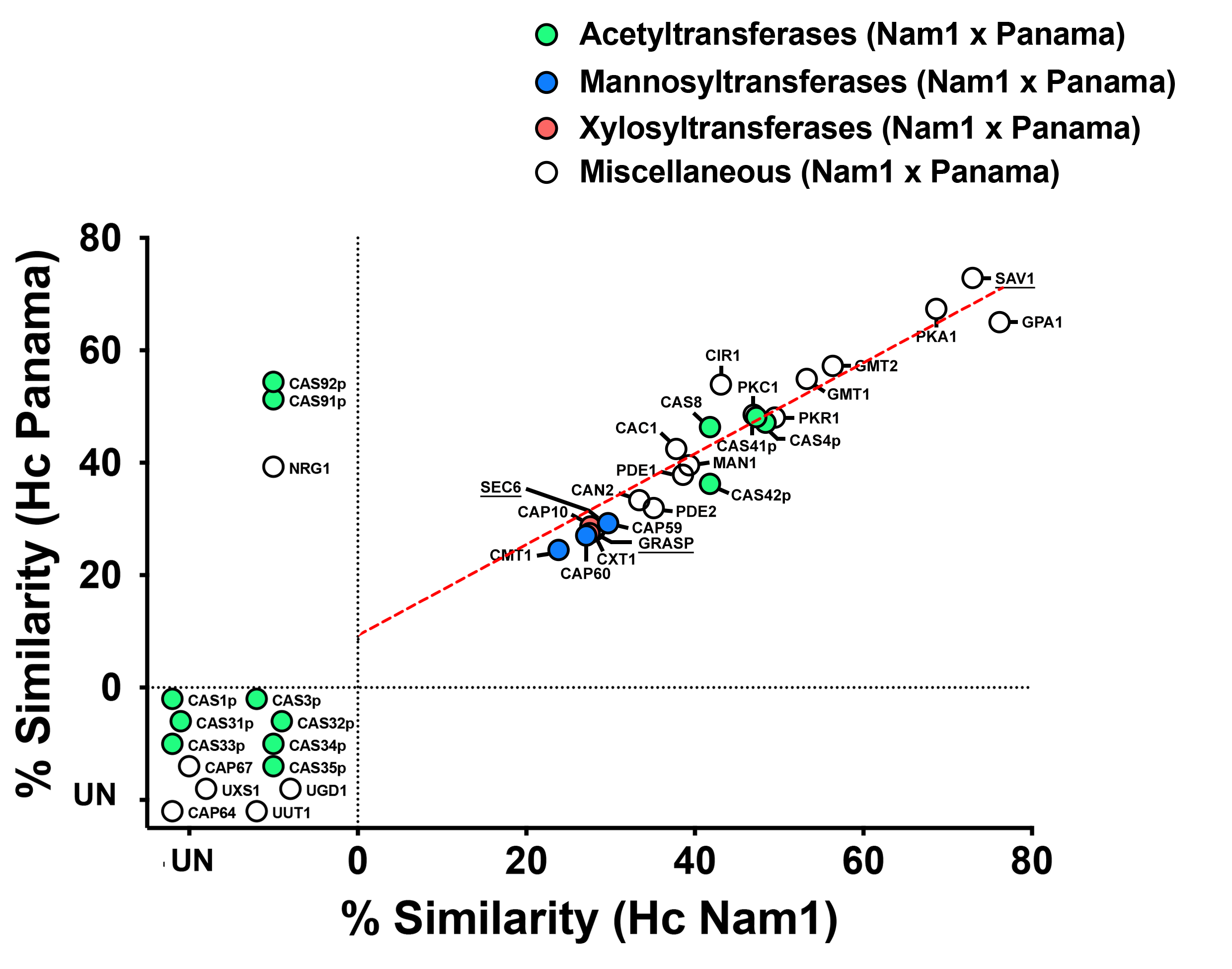

Supplement: Supplementary Figure 2 — Correlation the H. capsulatum Nam1/WU24 and the Panama/G186AR strain/H82 strain regarding their similarities to the C neoformans var. grubii serotype A (strain H99/ATCC 208821) strain. Circle filling colors denote the acetyltransferases (green), mannosyltransferases (blue), acetyltransferases (pink), and miscellaneous (white) groups. Similarity values of proteins from the two strains displayed a correlation (R2 = 0.70, ****p<0.0001; UN – unidentified in the database). [file Image_2.tif]
