# Supplementary material for: Histoplasma capsulatum Glycans From Distinct Genotypes Share Structural and Serological Similarities to Cryptococcus neoformans Glucuronoxylomannan
Source: Front Cell Infect Microbiol. 2021 Jan 8;10:565571. doi: 10.3389/fcimb.2020.565571 (PMC7874066; doi:10.3389/fcimb.2020.565571)
Supplement: Supplementary Table 1 — Glucuronoxylomannan synthesis and capsular production-related proteins of C. neoformans and their respective orthologs in H. capsulatum Nam1/WU24 and the Panama/G186AR strain/H82 strains. Cryptococcal proteins were classified as acatyltransferases, mannosyltransferases, xylosyltransferases, and miscellaneous, including polysaccharide transport and GXM export proteins. Underlined protein names indicate those C. neoformans proteins with orthologs in H. capsulatum, with identical protein domains/families as annotated by Interpro/Pfam. [file Table_1.docx]

| **Acetyltransferases** | | | | | | | | | | | | | |
| --- | --- | --- | --- | --- | --- | --- | --- | --- | --- | --- | --- | --- | --- |
| **Capsular genes** | **Organism/**  **Uniprot Entry** | | **Gene name** | **Length** | **Molecular Weight**  **(Da)** | **Identity**  **(%)** | **Score** | **E value** | **InterPro Predictions** | | **Pfam Predictions** | | |
|  |  |  |  |  |  |  |  |  | **Domain** | **Domain Residues** | | **Family** | **Family Residue** |
| **CAS1p** | *Cn* | **Q8X226** | **Probable O-acetyltransferase** CAS1  *Cryptococcus neoformans* var. *grubii* serotype A (strain H99 / ATCC 208821 / CBS 10515 / FGSC 9487) | 959 | 107,298 | - | - | - | Cas 1 10 TM Acyl Transferase  domain  (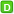IPR012419) | 381-820 | | Cas 1 10 TM Acyl Transferase domain  (PF07779) | 377-825 |
|  | *Hc* | N/F | - | - | - | - | - | - | - | - | | - | - |
| **CAS3p** | *Cn* | **J9VKK8** | Uncharacterized protein  *Cryptococcus neoformans* var. *grubii* serotype A (strain H99 / ATCC 208821 / CBS 10515 / FGSC 9487) | 500 | 56,297 | - | - | - | - | **-** | | - | **-** |
|  | *Hc* | **N/F** | - | - | - | - | - | - | - | - | | - | - |
| **CAS4p** | *Cn* | **J9VNE4** | TPT domain-containing protein  *Cryptococcus neoformans* var. *grubii* serotype A (strain H99 / ATCC 208821 / CBS 10515 / FGSC 9487) | 344 | 37,569 | - | - | - | Sugar-phosphate transporter domain  (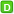IPR004853) | 55-338 | | Triose-phosphate transporter family  (PF03151) | 51-339 |
|  | *Hc* | A6R8J8 | **TPT domain-containing protein**  *Ajellomyces capsulatus* (strain NAm1 / WU24) | 274 | 30,202 | 48.5% | 238 | 4.9x10^-82^ | Sugar-phosphate transporter domain  (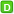IPR004853) | 12-265 | | Triose-phosphate transporter family  (PF03151) | 3-266 |
|  |  | C0NLA7 | **DUF250 domain-containing protein**  ***Ajellomyces capsulatus* (strain G186AR / H82 / ATCC MYA-2454 / RMSCC 2432)** | 325 | 35,466 | 47.3% | 216 | 3.6x10^-67^ | Sugar-phosphate transporter domain  (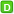IPR004853) | 33-253 | | Triose-phosphate transporter family  (PF03151) | 19-254 |
| **CAS8** | *Cn* | **J9VWY7** | Acetyl-CoA transporter  *Cryptococcus neoformans* var. *grubii* serotype A (strain H99 / ATCC 208821 / CBS 10515 / FGSC 9487) | 601 | 65,756 | - | - | - | AmpG-like permease/Acetyl-coenzyme A transporter 1  (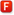IPR004752) | 84-597 | | - | - |
|  |  |  |  |  |  |  |  |  | Acetyl-coenzyme A transporter 1  (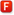IPR024371) | 95-305  309-597 | | Acetyl-coenzyme A transporter 1  (PF13000) | 94-311  303-597 |
|  |  |  |  |  |  |  |  |  | Major facilitator superfamily transporter  (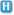IPR036259) | 91-579 | | - | - |
|  | *Hc* | C0NLV9 | Acetyl coenzyme A transporter  ***Ajellomyces capsulatus* (strain G186AR / H82 / ATCC MYA-2454 / RMSCC 2432)** | 703 | 77,212 | 46.5% | 462 | 1.3x10^-151^ | AmpG-like permease/Acetyl-coenzyme A transporter 1  (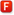IPR004752) | 204-696 | | - | - |
|  |  |  |  |  |  |  |  |  | Acetyl-coenzyme A transporter 1  (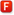IPR024371) | 218-427  514-695 | | Acetyl-coenzyme A transporter 1  (PF13000) | 217-431  423-515  509-696 |
|  |  |  |  |  |  |  |  |  | Major facilitator superfamily transporter  (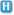IPR036259) | 205-680 | | - | - |
|  |  | A6RCB7 | **Uncharacterized protein**  *Ajellomyces capsulatus* (strain NAm1 / WU24) | 690 | 76,644 | 41.9% | 381 | 1.8×10^-72^ | AmpG-like permease/Acetyl-coenzyme A transporter 1  (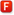IPR004752) | 306-404  408-683 | |  |  |
|  |  |  |  |  |  |  |  |  | Acetyl-coenzyme A transporter 1  (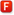IPR024371) | 320-406  411-498  501-682 | | Acetyl-coenzyme A transporter 1  (PF13000) | 319-411  405-502  496-683 |
|  |  |  |  |  |  |  |  |  | Major facilitator superfamily transporter  (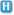IPR036259) | 313-668 | | - | - |
| **CAS31p** | *Cn* | J9VMB3 | Uncharacterized protein  *Cryptococcus neoformans* var. *grubii* serotype A (strain H99 / ATCC 208821 / CBS 10515 / FGSC 9487) | 508 | 56,707 | - | - | - | - | - | | - | - |
|  | *Hc* | - | **-** | - | - | - | - | - | - | - | | - | - |
| **CAS32p** | *Cn* | **J9VKP7** | **Uncharacterized protein**  *Cryptococcus neoformans* var. *grubii* serotype A (strain H99 / ATCC 208821 / CBS 10515 / FGSC 9487) | 551 | 62,415 | - | - | - | - | - | | - | - |
|  | *Hc* | - | **-** | - | - | - | - | - | - | - | | - | - |
| **CAS33p** | *Cn* | J9VKZ0 | Uncharacterized protein  *Cryptococcus neoformans* var. *grubii* serotype A (strain H99 / ATCC 208821 / CBS 10515 / FGSC 9487) | 603 | 67,356 | - | - | - | SGNH hydrolase-superfamily  (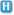IPR036514) | 161-357 | | - | - |
|  | *Hc* | - | - | - | - | - | - | - | - | - | | - | - |
| **CAS34p** | *Cn* | J9VYW9 | Uncharacterized protein  *Cryptococcus neoformans* var. *grubii* serotype A (strain H99 / ATCC 208821 / CBS 10515 / FGSC 9487) | 674 | 74,954 | - | - | - | - | - | | - | - |
|  | *Hc* | - | - | - | - | - | - | - | - | - | | - | - |
| **CAS35p** | *Cn* | J9VHN1 | Uncharacterized protein  *Cryptococcus neoformans* var. *grubii* serotype A (strain H99 / ATCC 208821 / CBS 10515 / FGSC 9487) | 628 | 67,909 | - | - | - | - | - | | - | - |
|  | *Hc* | - | - | - | - | - | - | - | - | - | | - | - |
| **CAS41p** | *Cn* | **J9VNC0** | TPT domain-containing protein  *Cryptococcus neoformans* var. *grubii* serotype A (strain H99 / ATCC 208821 / CBS 10515 / FGSC 9487) | 356 | 38,662 | - | - | - | Sugar phosphate transporter domain  (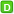IPR004853) | 65-350 | | Triose-phosphate transporter  family  (PF03151) | 63-351 |
|  | *Hc* | C0NLA7 | **DUF250 domain-containing protein**  *Ajellomyces capsulatus* (strain G186AR / H82 / ATCC MYA-2454 / RMSCC 2432) | 325 | 35,466 | 48.3% | 224 | 9.4×10^-65^ | Sugar phosphate transporter domain  (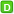IPR004853) | 33-253 | | Triose-phosphate transporter  family  (PF03151) | 19-254 |
|  |  | A6R8J8 | **TPT domain-containing protein**  *Ajellomyces capsulatus* (strain NAm1 / WU24) | 274 | 30,202 | 47.4% | 594 | 9.0×10^-78^ | Sugar phosphate transporter domain  (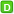IPR004853) | 12-265 | | Triose-phosphate transporter  family  (PF03151) | 3-266 |
| **CAS42p** | *Cn* | Q5WR58 | Cas42p  *Cryptococcus neoformans* var. neoformans | 539 | 60,519 | - | - | - | Sugar phosphate transporter domain  (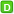IPR004853) | 96-393 | | Triose-phosphate transporter  family  (PF03151)) | 94-393 |
|  | *Hc* | A6RAA0 | **TPT domain-containing protein**  *Ajellomyces capsulatus* (strain NAm1 / WU24) | 447 | 48,341 | 41.9% | 493 | 6.0×10^-61^ | Sugar phosphate transporter domain  (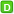IPR004853) | 59-301 | | Triose-phosphate transporter  family  (PF03151) | 27-301 |
|  |  | C0NSB8 | Solute carrier family 35 member C2 *Ajellomyces capsulatus* (strain G186AR / H82 / ATCC MYA-2454 / RMSCC 2432) | 663 | 71,600 | 36.4% | 504 | 5.0x10^-61^ | Sugar phosphate transporter domain  (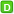IPR004853) | 210-451 | | Triose-phosphate transporter  family  (PF03151) | 166-451 |
| **CAS91p** | *Cn* | J9VIK7 | Maltose O-acetyl transferase  *Cryptococcus neoformans* var. *grubii* serotype A (strain H99 / ATCC 208821 / CBS 10515 / FGSC 9487) | 213 | 23,282 | - | - | - | Maltose/galactoside acetyltransferase  (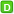IPR024688) | 26-77 | | - | - |
|  |  |  |  |  |  |  |  |  | Trimeric LpxA-like superfamily  (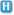IPR011004) | 64-207 | | - | - |
|  |  |  |  |  |  |  |  |  | Hexapeptide repeat  (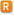IPR001451) | 151-185 | | Bacterial transferase hexapeptide (six repeats)  (PF00132) | 151-185 |
|  | *Hc* | C0NM49 | Acetyltransferase  *Ajellomyces capsulatus* (strain G186AR / H82 / ATCC MYA-2454 / RMSCC 2432) | 220 | 23,969 | 51.3% | 128 | 1.0x10^-41^ | Maltose/galactoside acetyltransferase  (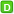IPR024688) | 7-61 | | Maltose acetyltransferase  (PF12464) | 9-60 |
|  |  |  |  |  |  |  |  |  | Trimeric LpxA-like superfamily  (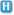IPR011004) | 4-211 | | - | - |
|  |  |  |  |  |  |  |  |  | Hexapeptide repeat  (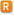IPR001451) | 152-186 | | Bacterial transferase hexapeptide (six repeats)  (PF00132) | 151-186 |
|  |  |  |  |  |  |  |  |  | Hexapeptide transferase, conserved site  (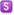IPR018357) | 160-188 | | - | - |
| **CAS92p** | *Cn* | J9VU56 | **Maltose O-acetyltransferase**  *Cryptococcus neoformans* var. *grubii* serotype A (strain H99 / ATCC 208821 / CBS 10515 / FGSC 9487) | 181 | 20,301 | - | - | - | Trimeric LpxA-like superfamily  (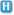IPR011004) | 73-143 | | - | - |
|  |  |  |  |  |  |  |  |  | Hexapeptide repeat  (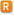IPR001451) | 105-139 | | - | - |
|  |  |  |  |  |  |  |  |  | Hexapeptide transferase, conserved site  (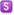IPR018357) | 113-141 | | Bacterial transferase hexapeptide (six repeats)  (PF00132) | 104-139 |
|  | *Hc* | C0NM49 | Acetyltransferase  *Ajellomyces capsulatus* (strain G186AR / H82 / ATCC MYA-2454 / RMSCC 2432) | 220 | 23,969 | 54.4% | 98 | 5.0×10^--23^ | Maltose/galactoside acetyltransferase  (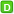IPR024688) | 7-61 | | Maltose acetyltransferase  (PF12464) | 9-60 |
|  |  |  |  |  |  |  |  |  | Trimeric LpxA-like superfamily  (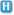IPR011004) | 4-211 | | - | - |
|  |  |  |  |  |  |  |  |  | Hexapeptide repeat  (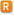IPR001451) | 152-186 | | Bacterial transferase hexapeptide (six repeats)  (PF00132) | 151-186 |
|  |  |  |  |  |  |  |  |  | Hexapeptide transferase, conserved site  (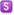IPR018357) | 160-188 | | - | - |
|  | | | | | | | | | | | | | |
| **Mannosyltransferase** | | | | | | | | | | | | | |
| **Capsular genes** | **Organism/**  **Uniprot Entry** | | **Gene name** | **Length** | **Molecular Weight**  **(Da)** | **Identity**  **(%)** | **Score** | **E value** | **InterPro Predictions** | | | **Pfam Predictions** | |
|  |  |  |  |  |  |  |  |  | **Domain** | **Domain Residues** | | **Family** | **Family Residue** |
| **CAP59** | *Cn* | J9VHJ1 | Alpha-1,3-mannosyltransferase  *Cryptococcus neoformans* var. *grubii* serotype A (strain H99 / ATCC 208821 / CBS 10515 / FGSC 9487) | 525 | 59,912 | - | - | - | Mannosyltransferase-1, CMT-1  (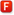IPR021047) | 9-524 | | Cryptococcal mannosyltransferase 1  (PF11735) | 161-397 |
|  | *Hc* | A6RGP6 | **Uncharacterized protein**  *Ajellomyces capsulatus* (strain NAm1 / WU24) | 432 | 48,608 | 29.8% | 130 | 2.0x10^-26^ | Mannosyltransferase-1, CMT-1  (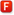IPR021047) | 27-401 | | Cryptococcal mannosyltransferase 1  (PF11735) | 77-328 |
|  |  | C0NZC4 | Uncharacterized protein *Ajellomyces capsulatus* (strain G186AR / H82 / ATCC MYA-2454 / RMSCC 2432) | 423 | 48,293 | 29.4% | 112 | 7.0x10^-26^ | Mannosyltransferase-1, CMT-1  (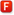IPR021047) | 48-423 | | Cryptococcal mannosyltransferase 1  (PF11735) | 49-300 |
| **CAP60** | *Cn* | J9VM07 | Capsular associated protein  *Cryptococcus neoformans* var. *grubii* serotype A (strain H99 / ATCC 208821 / CBS 10515 / FGSC 9487) | 593 | 66,784 | - | - | - | Mannosyltransferase-1, CMT-1  (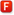IPR021047) | 158-381 | | Cryptococcal mannosyltransferase 1  (PF11735) | 157-392 |
|  | *Hc* | A6RGP6 | **Uncharacterized protein**  *Ajellomyces capsulatus* (strain NAm1 / WU24) | 432 | 48,608 | 27.2% | 74.3 | 4.0×10^-9^ | Mannosyltransferase-1, CMT-1  (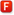IPR021047) | 27-401 | | Cryptococcal mannosyltransferase 1  (PF11735) | 77-328 |
|  |  | C0NZC4 | Uncharacterized protein *Ajellomyces capsulatus* (strain G186AR / H82 / ATCC MYA-2454 / RMSCC 2432) | 423 | 48,293 | 27.2% | 89.7 | 7.0x10^-9^ | Mannosyltransferase-1, CMT-1  (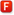IPR021047) | 48-423 | | Cryptococcal mannosyltransferase 1  (PF11735) | 49-300 |
| **CMT1** | *Cn* | J9VUY5 | Alpha-1,3-mannosyltransferase CMT1  *Cryptococcus neoformans* var. *grubii* serotype A (strain H99 / ATCC 208821 / CBS 10515 / FGSC 9487) | 465 | 53,213 | - | - | - | Mannosyltransferase-1, CMT-1  (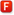IPR021047) | 127-375 | | Cryptococcal mannosyltransferase 1  (PF11735) | 127-375 |
|  | *Hc* | C0NZC4 | Uncharacterized protein *Ajellomyces capsulatus* (strain G186AR / H82 / ATCC MYA-2454 / RMSCC 2432) | 423 | 48,293 | 24.6% | 56.2 | 7.0x10^-13^ | Mannosyltransferase-1, CMT-1  (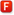IPR021047) | 48-423 | | Cryptococcal mannosyltransferase 1  (PF11735) | 49-300 |
|  |  | A6RGP6 | **Uncharacterized protein**  ***Ajellomyces capsulatus* (strain NAm1 / WU24)** | 432 | 48,608 | 23.9% | 57.8 | 2.2×10^-13^ | Mannosyltransferase-1, CMT-1  (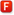IPR021047) | 78-328 | | Cryptococcal mannosyltransferase 1  (PF11735) | 77-328 |
|  | | | | | | | | | | | | | |
| **Xylosyltransferase** | | | | | | | | | | | | | |
| **Capsular genes** | **Organism/**  **Uniprot Entry** | | **Gene name** | **Length** | **Molecular Weight**  **(Da)** | **Identity**  **(%)** | **Score** | **E value** | **InterPro Predictions** | | | **Pfam Predictions** | |
|  |  |  |  |  |  |  |  |  | **Domain** | **Domain Residues** | | **Family** | **Family Residue** |
| **CAP10** | Cn | J9VN83 | **Capsular associated protein**  ***Cryptococcus neoformans* var. *grubii* serotype A (strain H99 / ATCC 208821 / CBS 10515 / FGSC 9487)** | 640 | 73,107 | - | - | - | Glycosyl transferase CAP10 domain  (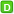IPR006598) | 299-604 | | Glycosyl transferase family 90  (PF05686) | 327-561 |
|  | Hc | C0NUD6 | **CAP10 domain-containing protein**  ***Ajellomyces capsulatus* (strain G186AR / H82 / ATCC MYA-2454 / RMSCC 2432)** | 785 | 89,224 | 28.0% | 206 | 1.6×10^-61^ | Glycosyl transferase cap10 domain  (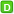IPR006598) | 501-777 | | Glycosyl transferase family 90  (PF05686) | 503-779 |
|  |  | A6QS63 | **CAP10 domain-containing protein**  ***Ajellomyces capsulatus* (strain NAm1 / WU24)** | 648 | 74,498 | 27.7% | 212 | 1.0×10^-58^ | Glycosyl transferase cap10 domain  (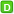IPR006598) | 380-648 | | Glycosyl transferase family 90  (PF05686) | 382-648 |
| **CXT1** | Cn | **J9VJM8** | **Beta-1,2 xylosyltransferase 1**  ***Cryptococcus neoformans* var. *grubii* serotype A (strain H99 / ATCC 208821 / CBS 10515 / FGSC 9487)** | 674 | 76,807 | - | - | - | Glycosyl transferase cap10 domain  (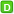IPR006598) | 383-659 | | Glycosyl transferase family 90  (PF05686) | 425-667 |
|  | Hc | C0NUD6 | **CAP10 domain-containing protein**  ***Ajellomyces capsulatus* (strain G186AR / H82 / ATCC MYA-2454 / RMSCC 2432)** | 785 | 89,224 | 27.6% | 210 | 1.6×10^-56^ | Glycosyl transferase cap10 domain  (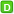IPR006598) | 510-777 | | Glycosyl transferase family 90  (PF05686) | 503-779 |
|  |  | A6QS63 | **CAP10 domain-containing protein**  ***Ajellomyces capsulatus* (strain NAm1 / WU24)** | 648 | 74,498 | 27.6% | 202 | 4.1×10^-54^ | Glycosyl transferase cap10 domain  (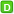IPR006598) | 380-648 | | Glycosyl transferase family 90  (PF05686) | 382-648 |
|  | | | | | | | | | | | | | |
| **Miscellaneous** | | | | | | | | | | | | | |
| **Capsular genes** | **Organism/**  **Uniprot Entry** | | **Gene name** | **Length** | **Molecular Weight**  **(Da)** | **Identity**  **(%)** | **Score** | **E value** | **InterPro Predictions** | | | **Pfam Predictions** | |
|  |  |  |  |  |  |  |  | **Domain** | **Domain Residues** | **Family** | | **Family Residue** |  |
| **CAP64** | *Cn* | J9VK40 | Capsular associated protein  ***Cryptococcus neoformans* var. *grubii* serotype A (strain H99 / ATCC 208821 / CBS 10515 / FGSC 9487)** | 956 | 103,221 | - | - | - | - | - | | - | - |
|  | *Hc* | N/F | - | - | - | - | - | - | - | - | | - | - |
| **CAP67** | *Cn* | P82476 | Chitin deacetylase  ***Cryptococcus neoformans* var. *grubii* serotype A (strain H99 / ATCC 208821 / CBS 10515 / FGSC 9487)** | 412 | 44,158 | - | - | - | NodB homology domain  (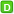IPR002509) | 120-314 | | Polysaccharide deacetilase  (PF01522) | 118-241 |
|  |  |  |  |  |  |  |  |  | Glycoside hydrolase/deacetylase, beta/alpha-barrel  (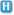IPR011330) | 118-322 | | - | - |
|  | *Hc* | N/F | - | - | - | - | - | - | - | - | | - | - |
| **CIR1** | *Cn* | **J9VSB5** | Iron regulator 1  ***Cryptococcus neoformans* var. *grubii* serotype A (strain H99 / ATCC 208821 / CBS 10515 / FGSC 9487)** | 952 | 102,811 | - | - | - | Transcription factor GATA  (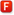IPR039355) | 2-865 | | - | - |
|  |  |  |  |  |  |  |  |  | Zinc finger, GATA-type  (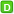IPR000679) | 302-357 | | GATA zinc finger  (PF00320) | 308-342 |
|  |  |  |  |  |  |  |  |  | Zinc finger, NHR/GATA-type  (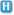IPR013088) | 300-358 | | - | - |
|  | *Hc* | **C0NNY1** | **Siderophore transcription factor SreA**  ***Ajellomyces capsulatus* (strain G186AR / H82 / ATCC MYA-2454 / RMSCC 2432)** | 630 | 67,234 | 54.1% | 244 | 1.0x10^-24^ | Transcription factor GATA  (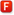IPR039355) | 94-582 | | - | - |
|  |  |  |  |  |  |  |  |  | Zinc finger, GATA-type  (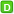IPR000679) | 122-175  285-340 | | GATA zinc finger  (PF00320) | 128-162  291-324 |
|  |  |  |  |  |  |  |  |  | Zinc finger, NHR/GATA-type  (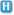IPR013088) | 116-172  283-343 | | - | - |
|  |  | **A6QU28** | **Uncharacterized protein *Ajellomyces capsulatus* (strain NAm1 / WU24)** | 630 | 65,575 | 43.2% | 251 | 3.0x10^-23^ | Transcription factor GATA  (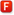IPR039355) | 87-575 | | - | - |
|  |  |  |  |  |  |  |  |  | Zinc finger, NHR/GATA-type  (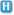IPR013088) | 116-172  283-343 | | - | - |
|  |  |  |  |  |  |  |  |  | Zinc finger, GATA-type  (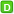IPR000679) | 128-159  291-323 | | GATA zinc finger  (PF00320) | 128-162  291-324 |
| **GPA1** | *Cn* | **J9VX74** | Guanine nucleotide-binding protein subunit alpha  ***Cryptococcus neoformans* var. *grubii* serotype A (strain H99 / ATCC 208821 / CBS 10515 / FGSC 9487)** | 432 | 47,800 | - | - | - | Guanine nucleotide binding protein (G-protein), alpha subunit  (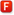IPR001019) | 40-431 | | G-protein alpha subunit  (PF00503) | 92-421 |
|  |  |  |  |  |  |  |  |  | Fungal G-protein, alpha subunit  (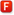IPR002975) | 138-150  265-275  355-367  382-393  408-418 | | - | - |
|  |  |  |  |  |  |  |  |  | G protein alpha subunit, helical insertion  (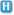IPR011025) | 140-261 | | - | - |
|  |  |  |  |  |  |  |  |  | P-loop containing nucleoside triphosphate hydrolase  (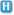IPR027417) | 110-429 | | - | - |
|  | *Hc* | **A6RFN8** | **Uncharacterized protein**  ***Ajellomyces capsulatus* (strain NAm1 / WU24)** | 93 | 10,693 | 76.3% | 170 | 9.0x10^-53^ | Guanine nucleotide binding protein (G-protein), alpha subunit  (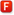IPR001019) | 1-92 | | G-protein alpha subunit  (PF00503) | 1-82 |
|  |  |  |  |  |  |  |  |  | P-loop containing nucleoside triphosphate hydrolase  (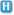IPR027417) | 110-429 | | - | - |
|  |  | **C0NRC3** | **Small G-protein GPA3**  ***Ajellomyces capsulatus* (strain G186AR / H82 / ATCC MYA-2454 / RMSCC 2432)** | 338 | 38,733 | 65.2% | 447 | 7.0x10^-162^ | Guanine nucleotide binding protein (G-protein), alpha subunit  (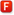IPR001019) | 3-337 | | G-protein alpha subunit  (PF00503) | 5-327 |
|  |  |  |  |  |  |  |  |  | G protein alpha subunit, helical insertion  (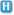IPR011025) | 33-154 | | - | - |
|  |  |  |  |  |  |  |  |  | P-loop containing nucleoside triphosphate hydrolase  (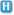IPR027417) | 10-331 | | - | - |
| **PKA1** | *Cn* | **J9VIW8** | **AGC/PKA protein kinase**  ***Cryptococcus neoformans* var. *grubii* serotype A (strain H99 / ATCC 208821 / CBS 10515 / FGSC 9487)** | 515 | 56,981 | - | - | - | Protein kinase domain  (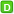IPR000719) | 203-459 | | Protein kinase domain  (PF00069) | 203-459 |
|  |  |  |  |  |  |  |  |  | AGC-kinase, C-terminal  (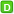IPR000961) | 460-515 | | - | - |
|  |  |  |  |  |  |  |  |  | Protein kinase-like domain superfamily  (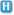IPR011009) | 193-496 | | - | - |
|  |  |  |  |  |  |  |  |  | Serine/threonine-protein kinase, active site  (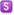IPR008271) | 322-334 | | - | - |
|  |  |  |  |  |  |  |  |  | Protein kinase, ATP binding site  (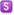IPR017441) | 209-232 | | - | - |
|  | *Hc* | **A6R2N1** | **cAMP-dependent protein kinase type 2**  ***Ajellomyces capsulatus* (strain NAm1 / WU24)** | 368 | 42,397 | 68.8% | 470 | 3.0x10^-156^ | Protein kinase domain  (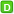IPR000719) | 57-312 | | Protein kinase domain  (PF00069) | 66-312 |
|  |  |  |  |  |  |  |  |  | AGC-kinase, C-terminal  (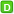IPR000961) | 313-368 | | - | - |
|  |  |  |  |  |  |  |  |  | Protein kinase-like domain superfamily  (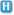IPR011009) | 67-337 | | - | - |
|  |  |  |  |  |  |  |  |  | Serine/threonine-protein kinase, active site  (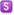IPR008271) | 176-188 | | - | - |
|  |  | **C0ND96** | **Protein kinase**  ***Ajellomyces capsulatus* (strain G186AR / H82 / ATCC MYA-2454 / RMSCC 2432)** | 450 | 52,150 | 67.6% | 488 | 7.0x10^-168^ | Protein kinase domain  (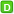IPR000719) | 139-394 | | Protein kinase domain  (PF00069) | 139-394 |
|  |  |  |  |  |  |  |  |  | AGC-kinase, C-terminal  (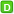IPR000961) | 395-450 | | - | - |
|  |  |  |  |  |  |  |  |  | Protein kinase-like domain superfamily  (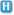IPR011009) | 123-418 | | - | - |
|  |  |  |  |  |  |  |  |  | Serine/threonine-protein kinase, active site  (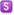IPR008271) | 258-270 | | - | - |
|  |  |  |  |  |  |  |  |  | Protein kinase, ATP binding site  (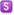IPR017441) | 145-168 | | - | - |
| **PKC1** | *Cn* | **J9W1R7** | **Protein kinase C**  ***Cryptococcus neoformans* var. *grubii* serotype A (strain H99 / ATCC 208821 / CBS 10515 / FGSC 9487)** | 1,086 | 120,768 | - | - | - | C2 domain  (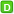IPR000008) | 248-347 | | C2 domain  (PF00168) | 248-349 |
|  |  |  |  |  |  |  |  |  | Protein kinase domain  (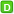IPR000719) | 757-1,016 | | Protein kinase domain  (PF00069) | 757-1,016 |
|  |  |  |  |  |  |  |  |  | AGC-kinase, C-terminal  (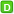IPR000961) | 1,017-1,086 | | - | - |
|  |  |  |  |  |  |  |  |  | Protein kinase C-like, phorbol ester/diacylglycerol-binding domain  (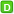IPR002219) | 450-500  517-570 | | Phorbol esters/diacylglycerol binding domain (C1 domain)  (PF00130) | 451-500  519-571 |
|  |  |  |  |  |  |  |  |  | HR1 rho-binding domain  (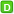IPR011072) | 6-73  157-222 | | Hr1 repeat  (PF02185) | 158-220 |
|  |  |  |  |  |  |  |  |  | Protein kinase, C-terminal  (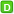IPR017892) | 1,039-1,076 | | Protein kinase C terminal domain  (PF00433) | 1,037-1,077 |
|  |  |  |  |  |  |  |  |  | Diacylglycerol/phorbol-ester binding  (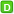IPR020454) | 448-462  464-473  475-486  557-569 | | - | - |
|  |  |  |  |  |  |  |  |  | Protein kinase C-like, HR1 domain  (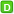IPR037312) | 149-220 | | - | - |
|  |  |  |  |  |  |  |  |  | Protein kinase C-like, C2 domain  (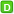IPR037778) | 250-361 | | - | - |
|  |  |  |  |  |  |  |  |  | Protein kinase-like domain superfamily  (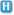IPR011009) | 754-1,069 | | - | - |
|  |  |  |  |  |  |  |  |  | C2 domain superfamily  (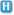IPR035892) | 245-360 | | - | - |
|  |  |  |  |  |  |  |  |  | HR1 repeat superfamily  (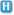IPR036274) | 152-219 | | - | - |
|  |  |  |  |  |  |  |  |  | Serine/threonine-protein kinase, active site  (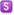IPR008271) | 878-890 | | - | - |
|  |  |  |  |  |  |  |  |  | Protein kinase, ATP binding site  (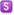IPR017441) | 763-786 | | - | - |
|  | *Hc* | **C0NFR3** | **Protein kinase C**  ***Ajellomyces capsulatus* (strain G186AR / H82 / ATCC MYA-2454 / RMSCC 2432)** | 1,125 | 125,286 | 48.7% | 996 | 0.0 | C2 domain  (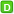IPR000008) | 248-345 | | C2 domain  (PF00168) | 248-355 |
|  |  |  |  |  |  |  |  |  | Protein kinase domain  (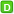IPR000719) | 800-1,059 | | Protein kinase domain  (PF00069) | 924-1,059 |
|  |  |  |  |  |  |  |  |  | AGC-kinase, C-terminal  (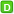IPR000961) | 1,060-1,125 | | - | - |
|  |  |  |  |  |  |  |  |  | Protein kinase C-like, phorbol ester/diacylglycerol-binding domain  (IPR002219) | 453-503  521-573 | | Phorbol esters/diacylglycerol binding domain (C1 domain)  (PF00130) | 454-504  522-574 |
|  |  |  |  |  |  |  |  |  | HR1 rho-binding domain  (IPR011072) | 6-69  157-225 | | Hr1 repeat  (PF02185) | 7-70  168-225 |
|  |  |  |  |  |  |  |  |  | Protein kinase, C-terminal  (IPR017892) | 1,080-1,120 | | Protein kinase C terminal domain  (PF00433) | 1,080-1,020 |
|  |  |  |  |  |  |  |  |  | Protein kinase C-like, C2 domain  (IPR037778) | 250-359 | | - | - |
|  |  |  |  |  |  |  |  |  | Protein kinase-like domain superfamily  (IPR011009) | 797-1,115 | | - | - |
|  |  |  |  |  |  |  |  |  | HR1 repeat superfamily  (IPR036274) | 168-226 | | - | - |
|  |  |  |  |  |  |  |  |  | Serine/threonine-protein kinase, active site  (IPR008271) | 921-933 | | - | - |
|  |  |  |  |  |  |  |  |  | Protein kinase, ATP binding site  (IPR017441) | 806-829 | | - | - |
|  |  | **A6QZ29** | **Protein kinase C**  ***Ajellomyces capsulatus* (strain NAm1 / WU24)** | 1,090 | 121,435 | 47.1% | 926 | 0.0 | C2 domain  (IPR000008) | 234-331 | | C2 domain  (PF00168) | 234-341 |
|  |  |  |  |  |  |  |  |  | Protein kinase domain  (IPR000719) | 765-1,024 | | Protein kinase domain  (PF00069) | 765-1,024 |
|  |  |  |  |  |  |  |  |  | AGC-kinase, C-terminal  (IPR000961) | 1,025-1,090 | | - | - |
|  |  |  |  |  |  |  |  |  | Protein kinase C-like, phorbol ester/diacylglycerol-binding domain  (IPR002219) | 439-489 | | Phorbol esters/diacylglycerol binding domain (C1 domain)  (PF00130) | 440-490 |
|  |  |  |  |  |  |  |  |  | HR1 rho-binding domain  (IPR011072) | 9-65  149-211 | | Hr1 repeat  (PF02185) | 7-66  152-211 |
|  |  |  |  |  |  |  |  |  | Protein kinase, C-terminal  (IPR017892) | 1,045-1,085 | | Protein kinase C terminal domain  (PF00433) | 1,045-1,085 |
|  |  |  |  |  |  |  |  |  | Protein kinase C-like, C2 domain  (IPR037778) | 236-345 | | - | - |
|  |  |  |  |  |  |  |  |  | Protein kinase-like domain superfamily  (IPR011009) | 762-1,080 | | - | - |
|  |  |  |  |  |  |  |  |  | HR1 repeat superfamily  (IPR036274) | 153-212 | | - | - |
|  |  |  |  |  |  |  |  |  | Serine/threonine-protein kinase, active site  (IPR008271) | 886-898 | | - | - |
|  |  |  |  |  |  |  |  |  | Protein kinase, ATP binding site  (IPR017441) | 771-794 | | - | - |
| **PKR1** | *Cn* | **J9VH50** | cAMP-dependent protein kinase regulatory subunit  ***Cryptococcus neoformans* var. *grubii* serotype A (strain H99 / ATCC 208821 / CBS 10515 / FGSC 9487)** | 482 | 52,519 | - | - | - | cAMP-dependent protein kinase regulatory subunit  (IPR012198) | 1-479 | | - | - |
|  |  |  |  |  |  |  |  |  | Cyclic nucleotide-binding domain  (IPR000595) | 219-351  353-473 | | Cyclic nucleotide-binding domain  (PF00027) | 237-336  371-459 |
|  |  |  |  |  |  |  |  |  | cAMP-dependent protein kinase regulatory subunit, dimerization-anchoring domain  (IPR003117) | 2-40 | | Regulatory subunit of type II PKA R-subunit  (PF02197) | 2-40 |
|  |  |  |  |  |  |  |  |  | RmlC-like jelly roll fold  (IPR014710) | 181-334  335-475 | | - | - |
|  |  |  |  |  |  |  |  |  | Cyclic nucleotide-binding-like  (IPR018490) | 198-343  337-465 | | - | - |
|  |  |  |  |  |  |  |  |  | Cyclic nucleotide-binding, conserved site  (IPR018488) | 246-262  298-315  380-396  421-438 | | - | - |
|  | *Hc* | **A6R780** | cAMP-dependent protein kinase regulatory subunit  ***Ajellomyces capsulatus* (strain NAm1 / WU24)** | 148 | 15,830 | 49.6% | 189 | 2.0×10^-36^ | Cyclic nucleotide-binding domain  (IPR000595) | 11-131 | | Cyclic nucleotide-binding domain  (PF00027) | 29-114 |
|  |  |  |  |  |  |  |  |  | RmlC-like jelly roll fold  (IPR014710) | 1-140 | | - | - |
|  |  |  |  |  |  |  |  |  | Cyclic nucleotide-binding-like  (IPR018490) | 2-122 | | - | - |
|  |  |  |  |  |  |  |  |  | Cyclic nucleotide-binding, conserved site  (IPR018488) | 38-54  78-95 | | - | - |
|  |  | **C0NGJ1** | cAMP-dependent protein kinase regulatory subunit  *Ajellomyces capsulatus* (strain G186AR / H82 / ATCC MYA-2454 / RMSCC 2432) | 451 | 47,979 | 48.2% | 290 | 1.0×10^-91^ | cAMP-dependent protein kinase regulatory subunit  (IPR012198) | 20-443 | | - | - |
|  |  |  |  |  |  |  |  |  | Cyclic nucleotide-binding domain  (IPR000595) | 276-312  334-416 | | Cyclic nucleotide-binding domain  (PF00027) | 199-297  332-417 |
|  |  |  |  |  |  |  |  |  | RmlC-like jelly roll fold  (IPR014710) | 136-295  296-442 | | - | - |
|  |  |  |  |  |  |  |  |  | Cyclic nucleotide-binding-like  (IPR018490) | 155-297  302-425 | | - | - |
|  |  |  |  |  |  |  |  |  | Cyclic nucleotide-binding, conserved site  (IPR018488) | 258-275  381-398 | | - | - |
| **PDE1** | *Cn* | J9VLM5 | Phosphodiesterase  ***Cryptococcus neoformans* var. *grubii* serotype A (strain H99 / ATCC 208821 / CBS 10515 / FGSC 9487)** | 480 | 52,551 | - | - | - | Cyclic-AMP phosphodiesterase, class-II  (IPR000396) | 12-463 | | cAMP phosphodiesterase, class-II  (PF02112) | 105-459 |
|  | *Hc* | A6QYF3 | **Uncharacterized protein**  *Ajellomyces capsulatus* (strain NAm1 / WU24) | 544 | 58,737 | 38.7% | 237 | 8.0×10^-13^ | Cyclic-AMP phosphodiesterase, class-II  (IPR000396) | 19-543 | | cAMP phosphodiesterase, class-II  (PF02112) | 25-385  476-539 |
|  |  |  |  |  |  |  |  |  | Ribonuclease Z/Hydroxyacylglutathione hydrolase-like  (IPR036866) | 115-375 | | - | - |
|  |  | C0NF15 | **Uncharacterized protein *Ajellomyces capsulatus* (strain G186AR / H82 / ATCC MYA-2454 / RMSCC 2432)** | 544 | 58,581 | 38.0% | 252 | 8.0×10^-13^ | Cyclic-AMP phosphodiesterase, class-II  (IPR000396) | 19-543 | | cAMP phosphodiesterase, class-II  (PF02112) | 25-385  476-539 |
|  |  |  |  |  |  |  |  |  | Ribonuclease Z/Hydroxyacylglutathione hydrolase-like  (IPR036866) | 116-375 | | - | - |
| **PDE2** | *Cn* | J9VKH8 | **Phosphodiesterase**  ***Cryptococcus neoformans* var. *grubii* serotype A (strain H99 / ATCC 208821 / CBS 10515 / FGSC 9487)** | 1,154 | 124,940 | - | - | - | 3'5'-cyclic nucleotide phosphodiesterase, catalytic domain  (IPR002073) | 644-1,011 | | 3'5'-cyclic nucleotide phosphodiesterase  (PF00233) | 718-998 |
|  |  |  |  |  |  |  |  |  | HD/PDEase domain  (IPR003607) | 716-938 | | - | - |
|  |  |  |  |  |  |  |  |  | 3'5'-cyclic nucleotide phosphodiesterase, catalytic domain superfamily  (IPR036971) | 642-1,011 | | - | - |
|  |  |  |  |  |  |  |  |  | 3'5'-cyclic nucleotide phosphodiesterase, conserved site  (IPR023174) | 793-804 | | - | - |
|  | *Hc* | A6R1R4 | **Phosphodiesterase**  ***Ajellomyces capsulatus* (strain NAm1 / WU24)** | 839 | 93,420 | 35.2% | 295 | 2.0×10^-42^ | 3'5'-cyclic nucleotide phosphodiesterase  (IPR023088) | 299-314  326-342  409-422  426-442 | | - | - |
|  |  |  |  |  |  |  |  |  | 3'5'-cyclic nucleotide phosphodiesterase, catalytic domain  (IPR002073) | 295-511 | | 3'5'-cyclic nucleotide phosphodiesterase  (PF00233) | 291-498 |
|  |  |  |  |  |  |  |  |  | HD/PDEase domain  (IPR003607) | 272-435 | | - | - |
|  |  |  |  |  |  |  |  |  | 3'5'-cyclic nucleotide phosphodiesterase, catalytic domain superfamily  (IPR036971) | 291-529 | | - | - |
|  |  |  |  |  |  |  |  |  | 3'5'-cyclic nucleotide phosphodiesterase, conserved site  (IPR023174) | 299-310 | | - | - |
|  | *Hc* | C0NC75 | **Phosphodiesterase**  ***Ajellomyces capsulatus* (strain G186AR / H82 / ATCC MYA-2454 / RMSCC 2432)** | 965 | 107,612 | 32.1% | 318 | 2.0×10^-63^ | 3'5'-cyclic nucleotide phosphodiesterase  (IPR023088) | 363-376  535-548  552-568 | | - | - |
|  |  |  |  |  |  |  |  |  | 3'5'-cyclic nucleotide phosphodiesterase, catalytic domain  (IPR002073) | 286-637 | | 3'5'-cyclic nucleotide phosphodiesterase  (PF00233) | 367-624 |
|  |  |  |  |  |  |  |  |  | HD/PDEase domain  (IPR003607) | 365-561 | | - | - |
|  |  |  |  |  |  |  |  |  | 3'5'-cyclic nucleotide phosphodiesterase, catalytic domain superfamily  (IPR036971) | 302-655 | | - | - |
|  |  |  |  |  |  |  |  |  | 3'5'-cyclic nucleotide phosphodiesterase, conserved site  (IPR023174) | 425-436 | | - | - |
| **CAC1** | *Cn* | **J9VV26** | Adenylate cyclase  ***Cryptococcus neoformans* var. *grubii* serotype A (strain H99 / ATCC 208821 / CBS 10515 / FGSC 9487)** | 2309 | 252,353 | - | - | - | Adenylyl cyclase class-3/4/guanylyl cyclase  (IPR001054) | 1,846-2,108 | | Adenylate and Guanylate cyclase catalytic domain  (PF00211) | 1,935-2,118 |
|  |  |  |  |  |  |  |  |  | PPM-type phosphatase domain  (IPR001932) | 1,586-1,880 | | Protein phosphatase 2C  (PF00481) | 1,612-1,873 |
|  |  |  |  |  |  |  |  |  | Adenylate cyclase G-alpha binding  (IPR013716) | 512-562 | | - | - |
|  |  |  |  |  |  |  |  |  | Nucleotide cyclase  (IPR029787) | 1,931-2,161 | | - | - |
|  |  |  |  |  |  |  |  |  | Leucine-rich repeat domain superfamily  (IPR032675) | 982-1,242  1,246-1,364  1,382-1,594 | | Leucine rich repeat  (PF13855) | 1,065-1,124  1,253-1,311  1,443-1,502 |
|  |  |  |  |  |  |  |  |  | PPM-type phosphatase domain superfamily  (IPR036457) | 1,578-1,882 | | - | - |
|  | *Hc* | **C0NCV3** | Adenylate cyclase  *Ajellomyces capsulatus* (strain G186AR / H82 / ATCC MYA-2454 / RMSCC 2432) | 1,699 | 188,077 | 42.6% | 1,020 | 3×10^-103^ | Ras-associating (RA) domain  (IPR000159) | 614-705 | | - | - |
|  |  |  |  |  |  |  |  |  | Adenylyl cyclase class-3/4/guanylyl cyclase  (IPR001054) | 1,168-1,450 | | Adenylate and Guanylate cyclase catalytic domain  (PF00211) | 1,276-1,456 |
|  |  |  |  |  |  |  |  |  | PPM-type phosphatase domain  (IPR001932) | 1,159-1,216 | | Protein phosphatase 2C  (PF00481) | 1,146-1,216 |
|  |  |  |  |  |  |  |  |  | Adenylate cyclase G-alpha binding  (IPR013716) | 518-566 | | Adenylate cyclase G-alpha binding domain  (PF08509) | 515-556 |
|  |  |  |  |  |  |  |  |  | Nucleotide cyclase  (IPR029787) | 1,276-1,512 | | - | - |
|  |  |  |  |  |  |  |  |  | Leucine-rich repeat domain superfamily  (IPR032675) | 706-866  867-977  978-1,098 | | Leucine-rich repeat  (PF13855) | 804-863 |
|  |  |  |  |  |  |  |  |  | PPM-type phosphatase domain superfamily  (IPR036457) | 1,120-1,225 | | - | - |
|  | *Hc* | A6R296 | **Uncharacterized protein**  *Ajellomyces capsulatus* (strain NAm1 / WU24) | 1,014 | 112,059 | 37.9% | 834 | 6×10^-159^ | Adenylyl cyclase class-3/4/guanylyl cyclase  (IPR001054) | 681-921 | | Adenylate and Guanylate cyclase catalytic domain  (PF00211) | 789-894 |
|  |  |  |  |  |  |  |  |  | PPM-type phosphatase domain  (IPR001932) | 448-736 | | Protein phosphatase 2C  (PF00481) | 474-729 |
|  |  |  |  |  |  |  |  |  | Nucleotide cyclase  (IPR029787) | 773-878 | | - | - |
|  |  |  |  |  |  |  |  |  | Leucine-rich repeat domain superfamily  (IPR032675) | 3-154  222-448 | | Leucine-rich repeat  (PF13855) | 257-317 |
|  |  |  |  |  |  |  |  |  | PPM-type phosphatase domain superfamily  (IPR036457) | 439-742 | | - | - |
|  |  |  |  |  |  |  |  |  | Leucine-rich repeat  (IPR001611) | 62-83  85-106  108-129  131-152  258-327  330-351  381-403 | | - | - |
|  |  |  |  |  |  |  |  |  | Leucine-rich repeat,  Typical subtype  (IPR003591) | 60-81  83-105  106-129  280-303  304-327  329-351 | | - | - |
| **CAN2** | *Cn* | J9VPA7 | **Carbonic anhydrase**  *Cryptococcus neoformans* var. grubii serotype A (strain H99 / ATCC 208821 / CBS 10515 / FGSC 9487) | 239 | 26,600 | - | - | - | Carbonic anhydrase  (IPR001765) | 56-223 | | Carbonic anhydrase  (PF00484) | 63-219 |
|  |  |  |  |  |  |  |  |  | Carbonic anhydrase superfamily  (IPR036874) | 17-234 | | - | - |
|  | *Hc* | C0NJW2 | Carbonic anhydrase  *Ajellomyces capsulatus* (strain G186AR / H82 / ATCC MYA-2454 / RMSCC 2432) | 228 | 25,408 | 33.5% | 120 | 2.0×10^-38^ | Carbonic anhydrase  (IPR001765) | 43-204 | | Carbonic anhydrase  (PF00484) | 50-200 |
|  |  |  |  |  |  |  |  |  | Carbonic anhydrase superfamily  (IPR036874) | 4-217 | | - | - |
|  |  |  |  |  |  |  |  |  | Carbonic anhydrase, prokaryotic-like, conserved site  (IPR015892) | 95-115 | | - | - |
|  |  | A6QW32 | **Carbonic anhydrase**  *Ajellomyces capsulatus* (strain NAm1 / WU24) | 221 | 24,564 | 33.5% | 117 | 4.0×10^-37^ | Carbonic anhydrase  (IPR001765) | 36-197 | | Carbonic anhydrase  (PF00484) | 43-193 |
|  |  |  |  |  |  |  |  |  | Carbonic anhydrase superfamily  (IPR036874) | 3-211 | | - | - |
|  |  |  |  |  |  |  |  |  | Carbonic anhydrase, prokaryotic-like, conserved site  (IPR015892) | 88-108 | | - | - |
| **NRG1** | *Cn* | J9VJ62 | Transcriptional regulator Nrg1  *Cryptococcus neoformans* var. *grubii* serotype A (strain H99 / ATCC 208821 / CBS 10515 / FGSC 9487) | 359 | 38,090 | - | - | - | Zinc finger C2H2-type  (IPR013087) | 95-122  123-147 | | Zinc finger, C2H2 type  (PF00096) | 95-117  123-147 |
|  |  |  |  |  |  |  |  |  | Zinc finger C2H2 superfamily  (IPR036236) | 93-147 | | - | - |
|  | *Hc* | **C0NLF3** | **DNA-binding protein creA *Ajellomyces capsulatus* (strain G186AR / H82 / ATCC MYA-2454 / RMSCC 2432)** | 410 | 45,009 | 39.3% | 112 | 4.0×10^-17^ | Zinc finger C2H2-type  (IPR013087) | 31-58  59-88 | | - | - |
|  |  |  |  |  |  |  |  |  | Zinc finger C2H2 superfamily  (IPR036236) | 29-83 | | - |  |
| **MAN1** | *Cn* | **J9VS07** | Mannose-6-phosphate isomerase  *Cryptococcus neoformans* var. *grubii* serotype A (strain H99 / ATCC 208821 / CBS 10515 / FGSC 9487) | 434 | 47,771 | - | - | - | Mannose-6-phosphate isomerase, type I  (IPR001250) | 5-420 | | Phosphomannose isomerase type I  (PF01238) | 5-399 |
|  |  |  |  |  |  |  |  |  | Mannose-6-phosphate isomerase  (IPR016305) | 2-434 | | - | - |
|  |  |  |  |  |  |  |  |  | RmlC-like cupin domain superfamily  (IPR011051) | 3-433 | | - | - |
|  |  |  |  |  |  |  |  |  | RmlC-like jelly roll fold  (IPR014710) | 5-430 | | - | - |
|  |  |  |  |  |  |  |  |  | Phosphomannose isomerase, type I, conserved site  (IPR018050) | 128-136  291-316 | | - | - |
|  | *Hc* | **C0NRE7** | **Mannose-6-phosphate isomerase *Ajellomyces capsulatus* (strain G186AR / H82 / ATCC MYA-2454 / RMSCC 2432)** | 462 | 51,021 | 39.7% | 288 | 2.0×10^-97^ | Mannose-6-phosphate isomerase, type I  (IPR001250) | 5-432 | | Phosphomannose isomerase type I  (PF01238) | 5-409 |
|  |  |  |  |  |  |  |  |  | Mannose-6-phosphate isomerase  (IPR016305) | 3-455 | | - | - |
|  |  |  |  |  |  |  |  |  | RmlC-like cupin domain superfamily  (IPR011051) | 4-450 | | - | - |
|  |  |  |  |  |  |  |  |  | RmlC-like jelly roll fold  (IPR014710) | 5-447 | | - | - |
|  |  |  |  |  |  |  |  |  | Phosphomannose isomerase, type I, conserved site  (IPR018050) | 291-316 | | - | - |
|  |  | **A6RFM0** | **Mannose-6-phosphate isomerase *Ajellomyces capsulatus* (strain NAm1 / WU24)** | 462 | 50,951 | 39.4% | 283 | 2.0×10^-95^ | Mannose-6-phosphate isomerase, type I  (IPR001250) | 5-432 | | Phosphomannose isomerase type I  (PF01238) | 5-409 |
|  |  |  |  |  |  |  |  |  | Mannose-6-phosphate isomerase  (IPR016305) | 3-455 | | - | - |
|  |  |  |  |  |  |  |  |  | RmlC-like cupin domain superfamily  (IPR011051) | 4-450 | | - | - |
|  |  |  |  |  |  |  |  |  | RmlC-like jelly roll fold  (IPR014710) | 5-447 | | - | - |
|  |  |  |  |  |  |  |  |  | Phosphomannose isomerase, type I, conserved site  (IPR018050) | 126-134  291-316 | | - | - |
| **GMT1** | *Cn* | **J9VRU7** | GDP-mannose transporter 1  *Cryptococcus neoformans* var. *grubii* serotype A (strain H99 / ATCC 208821 / CBS 10515 / FGSC 9487) | 397 | 42,886 | - | - | - | UAA transporter  (IPR013657) | 69-335 | | UAA transporter family  (PF08449) | 60-343 |
|  |  |  |  |  |  |  |  |  | GDP-mannose transporter Vrg4-like  (IPR038736) | 36-383 | | - | - |
|  | *Hc* | C0NE64 | Golgi GDP-mannose transporter *Ajellomyces capsulatus* (strain G186AR / H82 / ATCC MYA-2454 / RMSCC 2432) | 382 | 42,122 | 55.1% | 397 | 6×10^-142^ | GDP-mannose transporter Vrg4-like  (IPR038736) | 33-356 | | - | - |
|  |  | A6R087 | Uncharacterized protein *Ajellomyces capsulatus* (strain NAm1 / WU24) | 289 | 32,264 | 53.4% | 289 | 5×10^-101^ | GDP-mannose transporter Vrg4-like  (IPR038736) | 33-271 | | - | - |
| **GMT2** | *Cn* | **J9VY61** | GDP-mannose transporter 2  *Cryptococcus neoformans* var. *grubii* serotype A (strain H99 / ATCC 208821 / CBS 10515 / FGSC 9487) | 421 | 45,309 | - | - | - | UAA transporter  (IPR013657) | 87-390 | | UAA transporter family  (PF08449) | 79-402 |
|  |  |  |  |  |  |  |  |  | GDP-mannose transporter Vrg4-like  (IPR038736) | 64-407 | | - | - |
|  | *Hc* | C0NE64 | Golgi GDP-mannose transporter *Ajellomyces capsulatus* (strain G186AR / H82 / ATCC MYA-2454 / RMSCC 2432) | 382 | 42,122 | 57.4% | 388 | 6×10^-138^ | GDP-mannose transporter Vrg4-like  (IPR038736) | 33-356 | | - | - |
|  |  | A6R087 | Uncharacterized protein *Ajellomyces capsulatus* (strain NAm1 / WU24) | 289 | 32,264 | 56.5% | 307 | 5×10^-97^ | GDP-mannose transporter Vrg4-like  (IPR038736) | 33-271 | | - | - |
| **UGD1** | *Cn* | **J9VR87** | UDP-glucose 6-dehydrogenase  *Cryptococcus neoformans* var. *grubii* serotype A (strain H99 / ATCC 208821 / CBS 10515 / FGSC 9487) | 468 | 51,396 | - | - | - | UDP-glucose/GDP-mannose dehydrogenase  (IPR017476) | 7-464 | | - | - |
|  |  |  |  |  |  |  |  |  | UDP-glucose 6-dehydrogenase, eukaryotic type  (IPR028356) | 1-468 | | - | - |
|  |  |  |  |  |  |  |  |  | UDP-glucose/GDP-mannose dehydrogenase, N-terminal  (IPR001732) | 8-190 | | UDP-glucose/GDP-mannose dehydrogenase family, NAD binding domain  (PF03721) | 7-198 |
|  |  |  |  |  |  |  |  |  | UDP-glucose/GDP-mannose dehydrogenase, dimerization  (IPR014026) | 216-310 | | UDP-glucose/GDP-mannose dehydrogenase family, central domain  (PF00984) | 216-311 |
|  |  |  |  |  |  |  |  |  | UDP-glucose/GDP-mannose dehydrogenase, C-terminal  (IPR014027) | 334-449 | | UDP-glucose/GDP-mannose dehydrogenase family, UDP binding domain  (PF03720) | 334-449 |
|  |  |  |  |  |  |  |  |  | 6-phosphogluconate dehydrogenase-like, C-terminal domain superfamily  (IPR008927) | 216-315 | | - | - |
|  |  |  |  |  |  |  |  |  | UDP-glucose/GDP-mannose dehydrogenase, C-terminal domain superfamily  (IPR036220) | 320-458 | | - | - |
|  |  |  |  |  |  |  |  |  | NAD(P)-binding domain superfamily  (IPR036291) | 7-220 | | UDP-glucose/GDP-mannose dehydrogenase family, NAD binding domain | 7-199 |
|  | *Hc* | N/F | - | - | - | - | - | - | - | - | | - | - |
| **UXS1** | *Cn* | **J9VQK9** | UDP-glucuronate decarboxylase  *Cryptococcus neoformans* var. *grubii* serotype A (strain H99 / ATCC 208821 / CBS 10515 / FGSC 9487) | 410 | 46,500 | - | - | - | NAD(P)-binding domain  (IPR016040) | 91-397 | | GDP-mannose 4,6 dehydratase  (PF16363) | 91-397 |
|  |  |  |  |  |  |  |  |  | UDP-glucuronate decarboxylase N-terminal  (IPR021761) | 4-77 | | UDP-glucuronate decarboxylase N-terminal  (PF11803) | 3-77 |
|  |  |  |  |  |  |  |  |  | NAD(P)-binding domain superfamily  (IPR036291) | 84-404 | | - | - |
|  | *Hc* | N/F | - | - | - | - | - | - | - | - | | - | - |
| **UUT1** | *Cn* | J9VW42 | UDP-Glucuronic Acid Transporter (TPT domain-containing protein)  *Cryptococcus neoformans* var. *grubii* serotype A (strain H99 / ATCC 208821 / CBS 10515 / FGSC 9487) | 550 | 58,322 | - | - | - | Sugar phosphate transporter domain  (IPR004853) | 247-530 | | Triose-phosphate Transporter family  (PF03151) | 244-533 |
|  | *Hc* | N/F | - | - | - | - | - | - | - | - | | - | - |
| **SAV1** | *Cn* | J9VKC4 | GTP-binding protein ypt2  *Cryptococcus neoformans* var. *grubii* serotype A (strain H99 / ATCC 208821 / CBS 10515 / FGSC 9487) | 211 | 23,473 | - | - | - | Small GTPase  (IPR001806) | 14-174 | | Ras family  (PF00071) | 14-175 |
|  |  |  |  |  |  |  |  |  | Small GTP-binding protein domain  (IPR005225) | 13-166 | |  |  |
|  |  |  |  |  |  |  |  |  | P-loop containing nucleoside triphosphate hydrolase  (IPR027417) | 8-184 | |  |  |
|  | *Hc* | A6RDF1 | GTP-binding protein SAS1  *Ajellomyces capsulatus* (strain NAm1 / WU24) | 205 | 22,778 | 73.1% | 304 | 3x10^-111^ | Small GTPase  (IPR001806) | 12-172 | | Ras family  (PF00071) | 12-173 |
|  |  |  |  |  |  |  |  |  | Small GTP-binding protein domain  (IPR005225) | 11-167 | | - | - |
|  |  |  |  |  |  |  |  |  | P-loop containing nucleoside triphosphate hydrolase  (IPR027417) | 6-177 | | - | - |
|  |  | C0NYV5 | GTP-binding protein SAS1  *Ajellomyces capsulatus* (strain G186AR / H82 / ATCC MYA-2454 / RMSCC 2432) | 205 | 22,778 | 73.1% | 304 | 3x10^-111^ | Small GTPase  (IPR001806) | 12-172 | | Ras family  (PF00071) | 12-173 |
|  |  |  |  |  |  |  |  |  | Small GTP-binding protein domain  (IPR005225) | 11-167 | | - | - |
|  |  |  |  |  |  |  |  |  | P-loop containing nucleoside triphosphate hydrolase  (IPR027417) | 6-177 | | - | - |
| **SEC6** | *Cn* | J9VUH1 | Uncharacterized protein  *Cryptococcus neoformans* var. *grubii* serotype A (strain H99 / ATCC 208821 / CBS 10515 / FGSC 9487) | 770 | 87,201 | - | - | - | Exocyst complex component EXOC3/Sec6  (IPR010326) | 8-743 | | Exocyst complex component Sec6  (PF06046) | 179-740 |
|  |  |  |  |  |  |  |  |  | Exocyst complex component EXOC3/Sec6, C-terminal domain  (IPR042532) | 569-743 | | - | - |
|  | *Hc* | A6RAW7 | Uncharacterized protein  *Ajellomyces capsulatus* (strain NAm1 / WU24) | 966 | 109,984 | 28.2% | 276 | 2.0x10^-84^ | Exocyst complex component EXOC3/Sec6  (IPR010326) | 233-965 | | Exocyst complex component Sec6  (PF06046) | 390-965 |
|  |  |  |  |  |  |  |  |  | Exocyst complex component EXOC3/Sec6, C-terminal domain  (IPR042532) | 795-965 | | - | - |
|  |  | C0NMW3 | Uncharacterized protein  *Ajellomyces capsulatus* (strain G186AR / H82 / ATCC MYA-2454 / RMSCC 2432) | 1,006 | 114,375 | 28.1% | 292 | 7.0x10^-90^ | Exocyst complex component EXOC3/Sec6  (IPR010326) | 258-1,005 | | Exocyst complex component Sec6  (PF06046) | 430-1005 |
|  |  |  |  |  |  |  |  |  | Exocyst complex component EXOC3/Sec6, C-terminal domain  (IPR042532) | 835-1,005 | | - | - |
| **GRASP** | *Cn* | J9VSZ0 | Uncharacterized protein  *Cryptococcus neoformans* var. *grubii* serotype A (strain H99 / ATCC 208821 / CBS 10515 / FGSC 9487) | 280 | 30,051 | - | - | - | GRASP55/65  (IPR007583) | 1-259 | | - | - |
|  |  |  |  |  |  |  |  |  | GRASP55/65 PDZ-like domain  (IPR024958) | 90-238 | | GRASP55/65 PDZ-like domain  (PF04495) | 5-116  90-236 |
|  |  |  |  |  |  |  |  |  | PDZ superfamily  (IPR036034) | 1-241 | | - | - |
|  | *Hc* | C0NCU2 | Uncharacterized protein  G186AR / H82 / ATCC MYA-2454 / RMSCC 2432) | 346 | 37,661 | 28.5% | 150 | 9.0x10^-30^ | GRASP55/65  (IPR007583) | 19-327 | | - | - |
|  |  |  |  |  |  |  |  |  | GRASP55/65 PDZ-like domain  (IPR024958) | 80-212 | | GRASP55/65 PDZ-like domain  (PF04495) | 80-214 |
|  |  |  |  |  |  |  |  |  | PDZ duperfamily  (IPR036034) | 16-115  118-215 | | - | - |
|  |  | A6R286 | Uncharacterized protein  *Ajellomyces capsulatus* (strain NAm1 / WU24) | 348 | 37,893 | 28.2% | 132 | 1.0x10^-29^ | GRASP55/65  (IPR007583) | 22-328 | | - | - |
|  |  |  |  |  |  |  |  |  | GRASP55/65 PDZ-like domain  (IPR024958) | 82-214 | | GRASP55/65 PDZ-like domain  (PF04495) | 82-216 |
|  |  |  |  |  |  |  |  |  | PDZ superfamily  (IPR036034) | 17-118  120-217 | | - | - |
